# Microbial inoculants with straw mediate degradation-level-specific changes in soil carbon cycling genes and microbial community

**DOI:** 10.1186/s40793-026-00898-4

**Published:** 2026-05-04

**Authors:** Yu Han, Jiaxin Cui, Xiantong Huang, Ping Guo, Shiqi Yang

**Affiliations:** Institute of Environment and Sustainable Development in Agriculture, Chinese Academy of Agricultural Sciences, Beijing, 100081 China

**Keywords:** Microbial inoculants, Straw, Degraded cinnamon soil, Soil C cycle genes, Microorganisms, Soil remediation

## Abstract

**Background:**

Enhancing soil organic carbon (SOC) sequestration in degraded lands is critical for climate mitigation and sustainable agriculture. While straw amendment combined with microbial inoculants holds great promise, the underlying mechanisms governing its impact on soil microbiome and carbon cycling genes remain poorly understood.

**Results:**

Here, we employed metagenomic sequencing to analyze responses in soil carbon (C) cycling genes, microbial community structure, and functional profiles across three degradation levels (severely, moderately, and non-degraded) of cinnamon soils under straw application alone or in combination with microbial inoculants. Results showed that both straw and straw-microbial inoculants treatments significantly improved soil properties, with improvements in available nitrogen and microbial biomass carbon (severe degradation), SOC (moderate degradation), and available nutrients (non-degradation). The combined application notably reshaped microbial communities by enhancing bacterial alpha diversity while reducing fungal diversity, and strengthened the relationship of relevant key soil C genes in severely degraded soils. Soil pH exhibited significant positive correlations with soil C cycling genes. Key bacterial genera (*Sphingomonas*, *Bradyrhizobium*) showed strong associations with ABC transporters and glycoside hydrolases, and fungal genus (*Chaetomium*) linked to pyruvate and purine metabolism. Importantly, we observed degradation-level specificity: straw addition significantly increased the abundance of the amylase gene K01214 (encoding α-amylase for starch hydrolysis) in severely degraded soils, whereas the straw-inoculant combination enriched the chitinase gene K01207 (encoding chitinase for chitin hydrolysis) in moderately degraded soils.

**Conclusions:**

Accordingly, we propose targeted application of straw with a customized chitinolytic-cellulolytic synthetic microbial community (1–5% of straw mass) to restore carbon cycling functions in degraded soils, while adopting optimized agronomic management to preserve microbiome stability in non-degraded soils. Our findings provide novel insights into microbial-mediated carbon cycling and a foundation for targeted soil restoration.

**Supplementary Information:**

The online version contains supplementary material available at 10.1186/s40793-026-00898-4.

## Background

Soil organic carbon (SOC) constitutes the largest terrestrial carbon pool, with a stock more than three times that of the atmospheric carbon reservoir [1]. The stability of SOC is critical for climate change mitigation, and its turnover processes play a decisive role in the global carbon cycle [2, 3]. Critically, the dynamics of this vast carbon pool are predominantly governed by soil microorganisms [4]. These microscopic engineers drive the decomposition, transformation, and stabilization of organic matter, thereby directly controlling the balance between carbon sequestration and release [5]. Consequently, the structure and functional capacity of soil microbial communities are fundamental determinants of both SOC dynamics and overall soil health. As a key indicator of soil quality, SOC content effectively reflects soil fertility status, with fertility improvement being recognized as the cornerstone of soil restoration [6, 7]. Previous research has demonstrated that total organic carbon (TOC) can serve as an early-warning indicator for soil degradation, where the 0.5% TOC represents a critical threshold in the degradation process [8]. Below this threshold, land degradation severely disrupts these essential microbially-mediated processes, impairs microbial community structure and function, and ultimately leads to a decline in soil fertility and ecosystem functionality [9]. Therefore, developing effective strategies to restore degraded soils must focus on rehabilitating the microbial drivers of SOC dynamics.

Cinnamon soils (classified as Luvisols and Cambisols) are a globally significant soil resource, covering approximately 240–315 million hectares worldwide, with about 25.16 million hectares located in China [10, 11]. Recognized for favorable agronomic properties including stable structure, balanced water retention, and inherent fertility, these soils are critical for global food security, underpinning approximately 20% of global cereal production and serving as a key production base for staple crops such as wheat and maize [12–14]. Beyond agriculture, cinnamon soils serve as significant carbon sinks and support key ecosystem functions, contributing to global biogeochemical cycles [15, 16]. In recent decades, rapid population growth has intensified land exploitation, accelerating soil degradation since the last century and posing severe threats to both ecological and food security [17]. To combat this degradation, previous studies have demonstrated the effectiveness of straw return, biochar application, and vegetation restoration in improving degraded soils, and have proposed underlying improvement mechanisms mainly from the perspectives of soil properties, enzyme activity, and microbial community assembly dynamics [18–21]. However, these mechanisms may be insufficient when applied to severely degraded soils, where the functional impairment of the native microbial community can critically limit the efficiency of straw decomposition and SOC stabilization, thereby constraining the overall rate and effectiveness of soil restoration.

Recent advances in microbial biotechnology have led to the development of microbial inoculants as a promising strategy for precisely modulating soil microbial communities to optimize key processes such as organic matter decomposition and nutrient cycling [22]. Current agronomic practices typically combine microbial inoculants with organic amendments or straw return within conventional fertilization regimes [23, 24]. These strategies leverage microbial catabolic pathways to enzymatically hydrolyze recalcitrant straw components (cellulose, hemicellulose, and lignin polymers) into bioavailable nutrients, thereby facilitating humification processes and enhancing soil fertility [25]. Notably, the decomposition efficiency varies substantially among microbial taxa, with certain cellulolytic *Bacillus* species and ligninolytic *Trichoderma* fungi demonstrating particularly high substrate specificity and enzymatic activity [26, 27]. A recent study has confirmed that composite fungal-bacterial inoculants can promote straw decomposition and nutrients release more effectively than single-strain inoculants [28]. However, the critical roles of straw incorporation coupled with microbial inoculants in shaping soil microbial community structure and biogeochemical cycling (particularly carbon) remain largely unclear [29]. A key uncertainty is how these effects vary with the initial degree of soil degradation and how they translate into shifts in the relevant functional gene networks. This knowledge gap significantly hinders both a mechanistic understanding of the remediation process and the development of optimized, site-specific restoration practices.

To address these critical knowledge gaps, we conducted a pot experiment using cinnamon soils categorized into three distinct degradation levels (severely, moderately, and non-degraded) based on soil organic matter (SOM) content, a key diagnostic indicator for soil degradation [30]. We applied treatments of straw alone and straw combined with a composite microbial inoculant (containing *Bacillus* and *Metarhizium* strains) to alfalfa. Metagenomic sequencing was utilized to investigate how varying degrees of soil degradation influence microbial community composition and C-cycling functional genes in cinnamon soils. The aims of this study were: (1) to examine the effects of straw and straw-microbial inoculants application on soil physicochemical properties and C-cycling gene profiles; (2) to assess their impacts on soil microbial community composition and diversity; (3) to elucidate how the interactions between straw and microbial inoculants modulate the functional potential of C-cycling genes across different degradation levels. This study elucidates microbiome-mediated restoration mechanisms in degraded cinnamon soils, specifically examining functional microbes responses to integrated organic-biological amendment strategies.

## Methods

### Experimental design

This study was conducted at the Jianping Experimental Station in Chaoyang, Liaoning Province (41°42′58′′ N, 119°35′47′′ E; 250 m altitude), a hilly region characterized by cinnamon soils and a semi-humid temperate continental monsoon climate with pronounced seasonal drought. The mean annual precipitation is 480 mm, with an average temperature of 7.9 °C and a frost-free period of 151 days. The area receives abundant sunshine, with annual sunshine duration ranging from 2,850 to 2,950 h. The farmland topsoil exhibits moderate sheet erosion, with severe degradation of the original humus layer and frequent exposure of the subsoil, indicating a severely degraded state. Soil erosion is the primary process driving cinnamon soil degradation in this region [30].

A pot experiment was conducted to investigate the effects of straw and microbial inoculants on cinnamon soils with varying degradation levels. Pots (25 cm in height and 20 cm in inner diameter) were each filled with 5 kg of air-dried topsoil (plow layer, sieved to 2 mm). During preparation, the soil was thoroughly mixed with pre-determined amendments before potting. The amendments included: (i) crushed maize straw (cut to 1 cm length) applied at 5% of the soil mass; and (ii) a composite microbial inoculant (supplied by Ningxia Zhongwei Taike Biotechnology Co., Ltd.) containing bacterial strains of *Bacillus subtilis* and fungal strains of *Metarhizium anisopliae*. According to the manufacturer, *B*. *subtilis* was selected for its efficacy in decomposing cellulose in straw [31], while *M*. *anisopliae* was included to promote plant growth and enhance plant resistance [32, 33]. The inoculant was applied at a dosage of 5% of the soil mass (i.e., 50 g per 1000 g of soil). This rate was selected as the upper limit of the manufacturer’s recommended field application range (1–5% w/w) for this composite product (Ningxia Zhongwei Taike Biotechnology Co., Ltd.), aiming to maximize the detectability of inoculant effects under controlled pot conditions. For treatments requiring the inoculant, the precise amount of inoculant powder was first uniformly blended with the corresponding portion of crushed straw to ensure even distribution, and this straw-inoculant mixture was then thoroughly incorporated into the bulk soil.

The experimental design was structured to answer two key questions across a soil degradation gradient. First, in severely degraded soil, we established a full set of controls (no amendment, straw alone, straw+inoculants) to assess the effects of each amendment. Second, for moderately and non-degraded soils, the design focused on the agronomic relevant question: given that straw is applied, does adding a microbial inoculant provide a significant additional benefit? Therefore, treatments for these soils compared “straw alone” versus “straw combined with inoculant”. This approach allowed us to evaluate how the efficacy of the inoculant as a straw enhancer varies with the initial degradation level of the soil. Accordingly, seven treatments were established in this study: SC (severely degraded soil, control), SS (severely degraded soil with straw addition), SI (severely degraded soil with straw combined with microbial inoculants), MS (moderately degraded soil with straw addition), MI (moderately degraded soil with straw combined with microbial inoculants), NS (non-degraded soil with straw addition), and NI (non-degraded soil with straw combined with microbial inoculants). Each treatment was replicated three times in a completely randomized design, with five alfalfa plants per pot. All pots were maintained in a greenhouse under natural sunlight and ambient temperature, with regular watering throughout the experimental period. All detailed methodologies, including protocols for inoculant quality control and pot experiment management, are provided in Appendix S1 of the Supplementary Materials.

### Soil sample collection and analysis

Soil samples were collected after straw decomposition and before alfalfa sowing using a five-point mixed sampling method, with 50–100 g of soil collected and homogenized from each pot. Each soil sample was sieved through a 2-mm mesh and then divided into two portions: one for the analysis of soil physicochemical properties and the other stored at -80 °C for subsequent soil DNA extraction.

More information on the soil physicochemical properties, including pH, soil organic carbon (SOC), alkali-hydrolyzable nitrogen (AN), available potassium (AK), available phosphorus (AP), and microbial biomass carbon (MBC), were described in previous study [12], and is provided in Appendix S2 of the Supplementary Materials.

### Soil DNA extraction and metagenome sequencing

Total genomic DNA was extracted from 0.25 g of frozen soil using the E.Z.N.A. Soil DNA Kit (Omega Bio-tek). Metagenomic sequencing was conducted on the Illumina HiSeq 2500 platform, generating approximately 5.0 Gb of 150-bp paired-end reads per sample. Bioinformatic analysis included quality filtering, de novo assembly with MEGAHIT [34], and functional annotation against the KEGG [35] and CAZy [36] databases to profile genes involved in soil C cycling. Comprehensive wet-lab and bioinformatic protocols are detailed in Appendix S3 of the Supplementary Materials.

### Statistical analysis

Data are presented as mean ± standard error. All statistical analyses and visualizations were conducted using R software (v4.1.3) and OriginPro (2024b). The normality of data distribution and homogeneity of variances were assessed using the Shapiro-Wilk test and Levene’s test, respectively. For data that satisfied both assumptions, one‑way analysis of variance (ANOVA) followed by Tukey’s HSD test was performed. Otherwise, the non‑parametric Kruskal–Wallis test followed by Dunn’s post hoc test was used. The significance level was set at *P* < 0.05. Differences in microbial community composition and function were examined using non-metric multidimensional scaling (NMDS) and permutational multivariate analysis of variance (PERMANOVA) in the “vegan” package [37–39]. The linear discriminant analysis effect size (LEfSe) method was employed to identify differentially abundant taxa (LDA score > 2.0) [30]. Co-occurrence networks of functional genes were constructed based on strong Spearman correlations (|r| > 0.6, *P* < 0.05) using the package “igraph” in R and visualized in Gephi (v 0.10.1) [40]. Relationships between soil properties, microbial taxa, and functional genes were evaluated using Pearson correlation and Mantel tests [41]. Functional predictions of differential C cycle genes were made using the package “DESeq2” for Statistical Analysis of Metagenomic Profiles (STAMP) [42].

## Results

### Soil properties

**Fig. 1:**
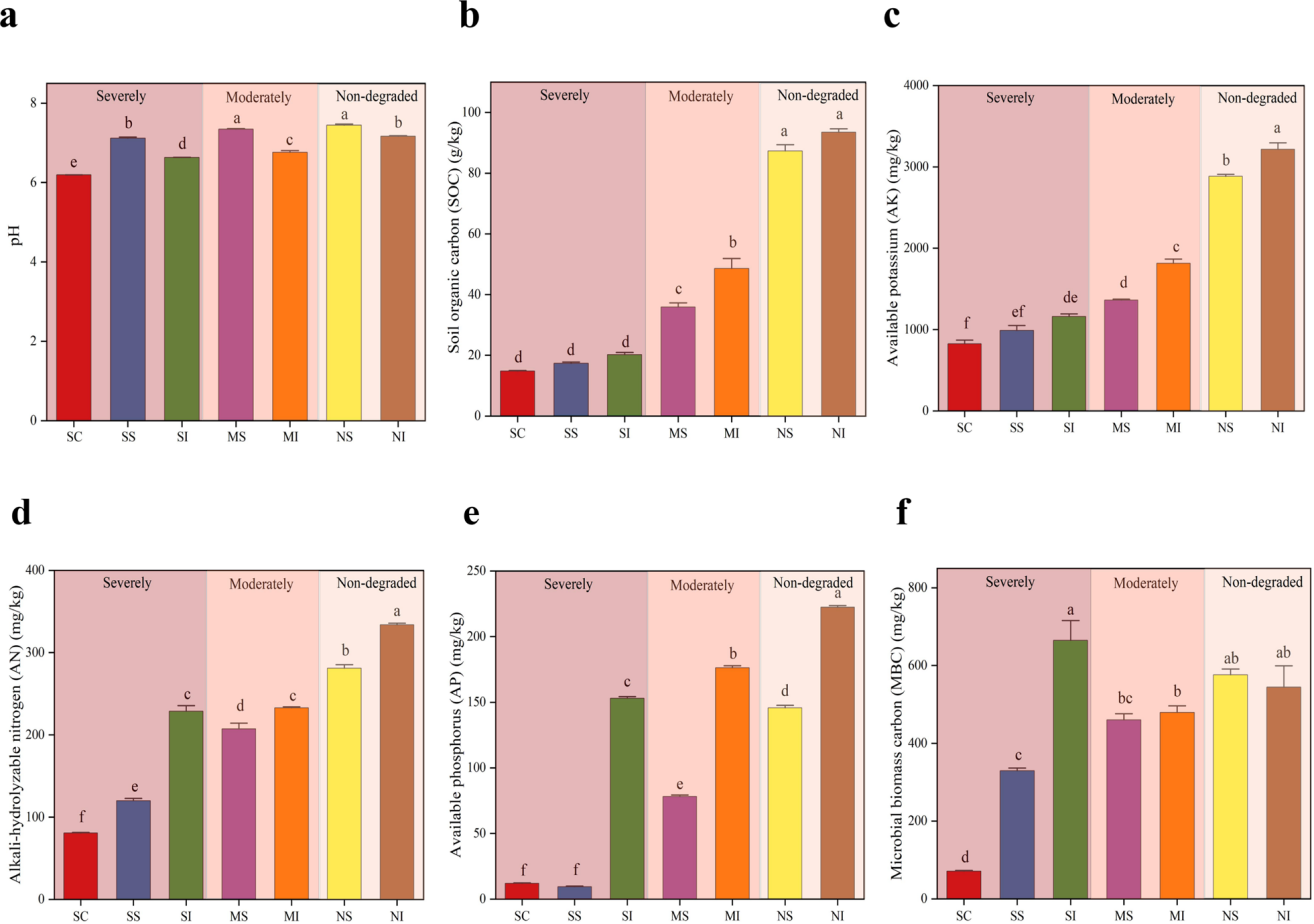
Effects of straw and microbial inoculant application on soil properties across different degradation levels of cinnamon soil. Sample data are presented as mean ± SE, *n* = 3. Different lowercase letters indicate significant differences (one-way ANOVA with Tukey’s HSD test, *P* < 0.05). **a** pH. **b** SOC, soil organic carbon. **c** AK, available potassium. **d** AN, alkali-hydrolyzable nitrogen. **e** AP, available phosphorus. **f** MBC, microbial biomass carbon. SC, severely degraded soil, control; SS, severely degraded soil with straw addition; SI, severely degraded soil with straw combined with microbial inoculants; MS, moderately degraded soil with straw addition; MI, moderately degraded soil with straw combined with microbial inoculants; NS, non-degraded soil with straw addition; NI, non-degraded soil with straw combined with microbial inoculants

Compared with the control (SC; severely degraded soil with no amendment), straw addition (SS; severely degraded soil) significantly increased the pH of severely degraded soil by 14.96%. However, the straw and microbial inoculants addition (SI; severely degraded soil) resulted in a significant 6.88% reduction in soil pH compared to SS (*P* < 0.05; Fig. 1a). In moderately degraded soil, the combined treatment (MI) showed a significant 7.94% decrease in pH compared to the straw-only treatment (MS). Similarly, in non-degraded soil, the combined treatment (NI) led to a significant 3.76% reduction in pH relative to the straw-only treatment (NS) (*P* < 0.05; Fig. 1a). Straw and microbial inoculants significantly enhanced SOC in moderately degraded soil by 35.50%, while no significant effect was observed in severely or non-degraded soils (Fig. 1b). Compared to the control, both straw-only (SS) and straw and microbial inoculants treatments (SI) markedly increased MBC in severely degraded soil, with increments of 362.83% and 833.98%, respectively (Fig. 1f). The inputs of straw and microbial inoculants significantly influenced the concentrations of available nutrients in soils with varying degradation levels. For instance, straw addition alone (SS) significantly elevated the AN content in severely degraded soil compared to the control (*P* < 0.05; Fig. 1d). The combined application of straw and microbial inoculants significantly enhanced available potassium (AK), AN, and available phosphorus (AP) in moderately and non-degraded soils compared to straw-only treatments (*P* < 0.05; Fig. 1).

### The community of soil C cycling genes

**Fig. 2:**
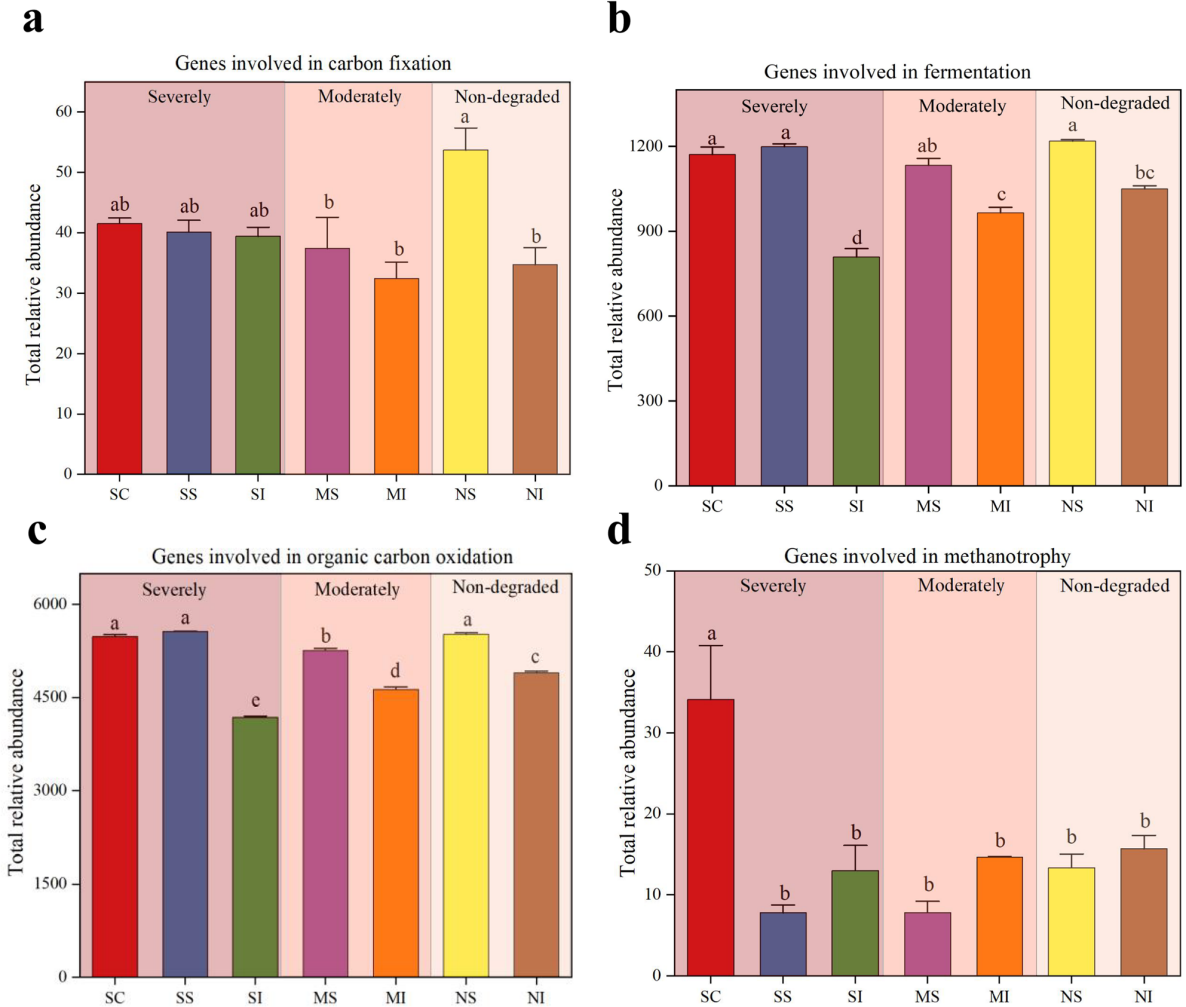
Total relative abundance of carbon (C)-cycling processes under straw and microbial inoculant application across soil degradation levels. **a** carbon fixation, **b** fermentation, **c** organic carbon oxidation and **d** methanotrophy. Sample data are presented as mean ± SE, *n* = 3. Different lowercase letters indicate significant differences (one-way ANOVA with Tukey’s HSD test, *P* < 0.05). SC, severely degraded soil, control; SS, severely degraded soil with straw addition; SI, severely degraded soil with straw combined with microbial inoculants; MS, moderately degraded soil with straw addition; MI, moderately degraded soil with straw combined with microbial inoculants; NS, non-degraded soil with straw addition; NI, non-degraded soil with straw combined with microbial inoculants

In severely and moderately degraded soils, neither straw application nor straw combined with microbial inoculants had a significant impact on carbon fixation gene abundance. However, in non-degraded soil, the straw-inoculants combination significantly reduced carbon fixation gene abundance compared to straw-only treatment (*P* < 0.05; Fig. 2a). Across all degradation levels, the combined application of straw and inoculants significantly decreased the abundance of genes associated with fermentation and organic carbon oxidation (*P* < 0.05; Fig. 2b, c). Compared to the control, all straw and straw-inoculants treatments markedly reduced the abundance of methanotrophy genes (*P* < 0.05; Fig. 2d).

**Fig. 3:**
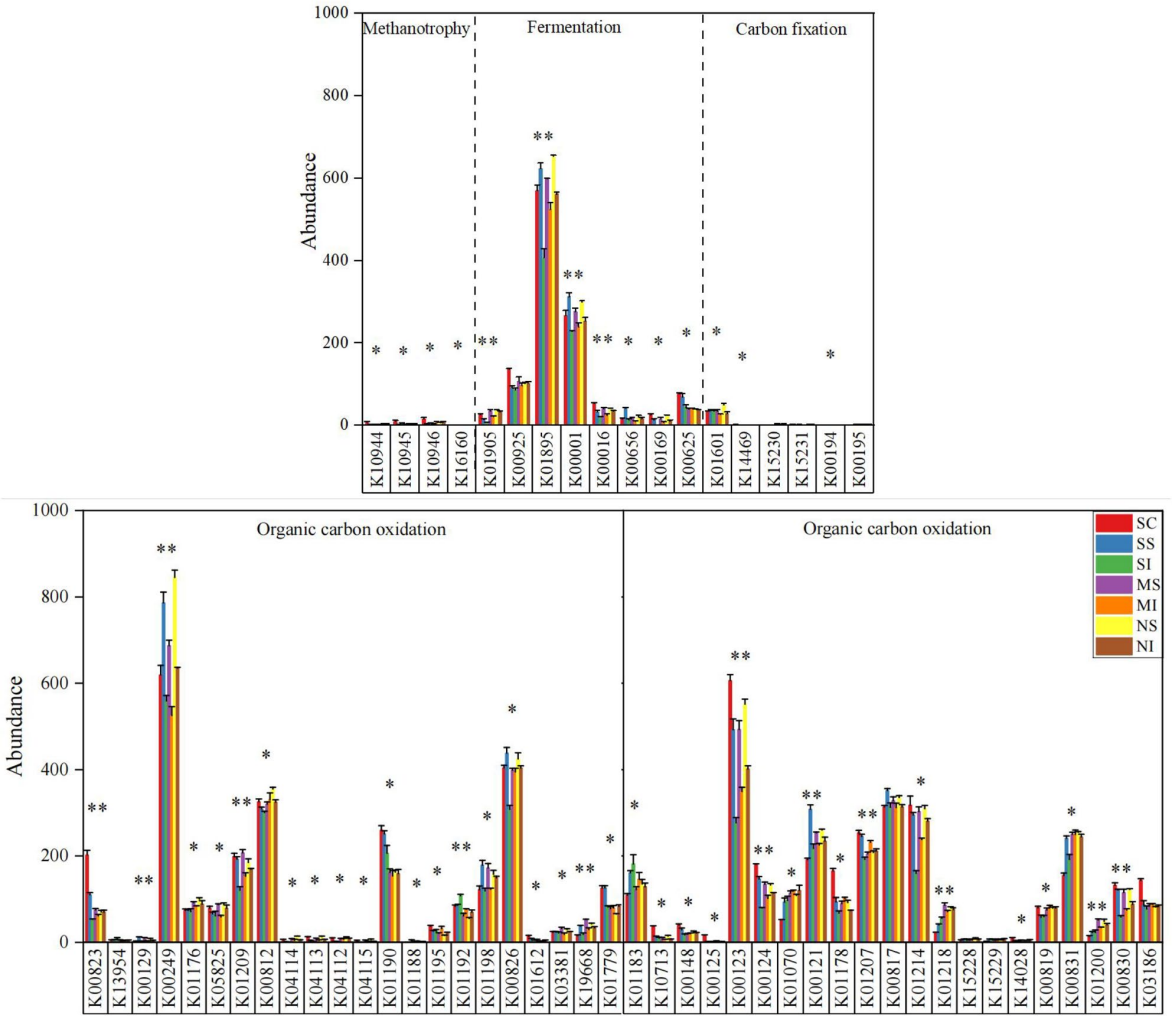
Abundance of C-cycling genes under straw and microbial inoculant application across soil degradation levels. Sample data are presented as mean ± SE, *n* = 3. The asterisk (*,**,***) indicates the significant (*P* < 0.05, *P* < 0.01, *P* < 0.001) differences among the seven treatments. SC, severely degraded soil, control; SS, severely degraded soil with straw addition; SI, severely degraded soil with straw combined with microbial inoculants; MS, moderately degraded soil with straw addition; MI, moderately degraded soil with straw combined with microbial inoculants; NS, non-degraded soil with straw addition; NI, non-degraded soil with straw combined with microbial inoculants

The K01895, K00249, K00826, and K00123 were the most dominant genes in all samples (Fig. 3). Compared to the control, straw-only treatments significantly increased the abundance of K01895 and K00249 (*P* < 0.05), whereas the straw-inoculants combination significantly reduced the abundance of K01895, K00249, and K00123 relative to straw alone (*P* < 0.05). For genes encoding organic carbon oxidation reactions, both straw and straw-inoculants treatments decreased the relative abundance of K00823 and K01190 in soils of all degradation levels (*P* < 0.05). Additionally, regarding fermentation-related genes, the straw-inoculants combination significantly reduced the abundance of K01895 (involved in acetate-to-acetyl-CoA conversion) and K00001 (associated with alcohol metabolism) compared to straw-only treatments across all degradation levels (*P* < 0.05).

### Responses of soil microbial diversity and community structure to amendments

#### Microbial diversity and compositions

**Fig. 4:**
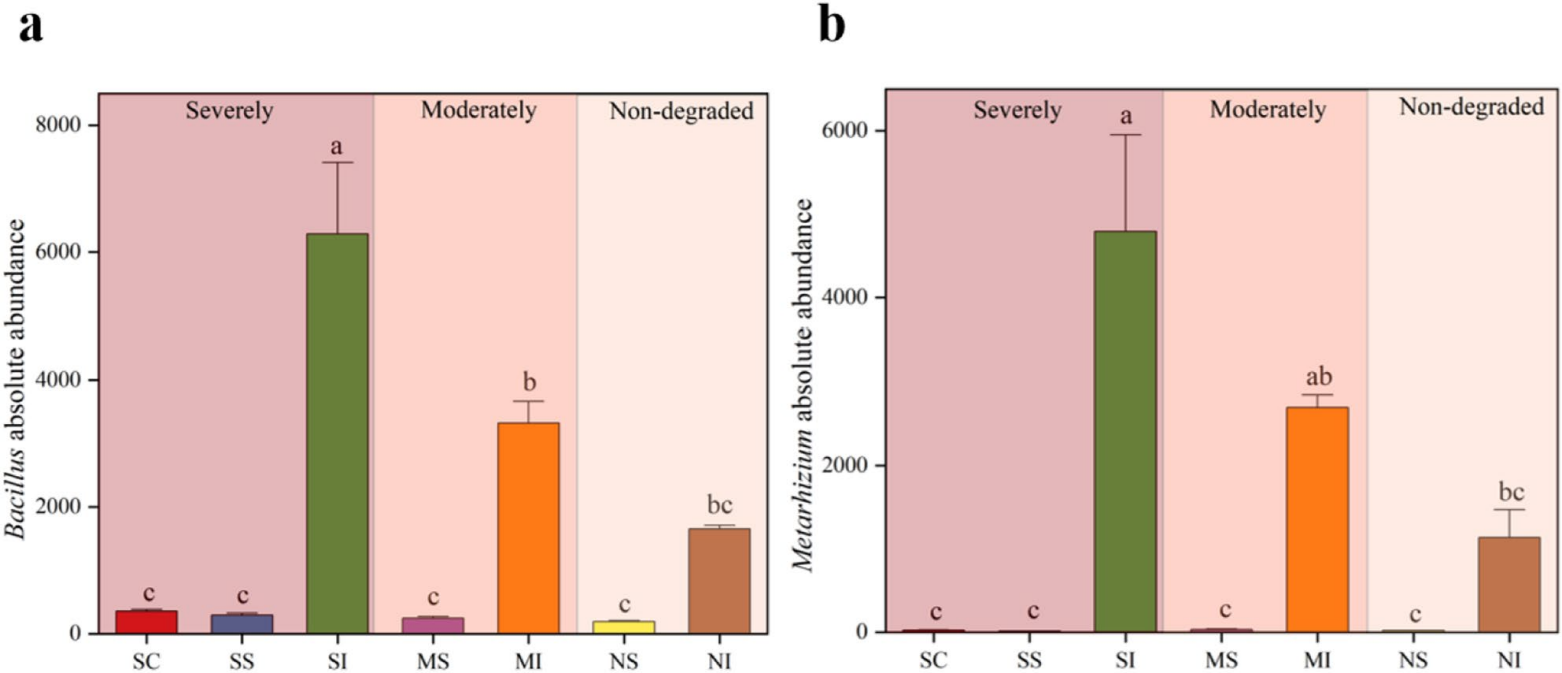
The accumulation of*Bacillus*(bacterial genus) and*Metarhizium*(fungi genus) in soils. **a***Bacillus* absolute abundance (number of reads); **b** *Metarhizium* absolute abundance (number of reads) in pot experiments (after 60 days of adding microbial inoculantts). Sample data are presented as mean ± SE, *n* = 3. Different lowercase letters indicate significant differences (one-way ANOVA with Tukey’s HSD test, *P* < 0.05). SC, severely degraded soil, control; SS, severely degraded soil with straw addition; SI, severely degraded soil with straw combined with microbial inoculants; MS, moderately degraded soil with straw addition; MI, moderately degraded soil with straw combined with microbial inoculants; NS, non-degraded soil with straw addition; NI, non-degraded soil with straw combined with microbial inoculants

**Fig. 5:**
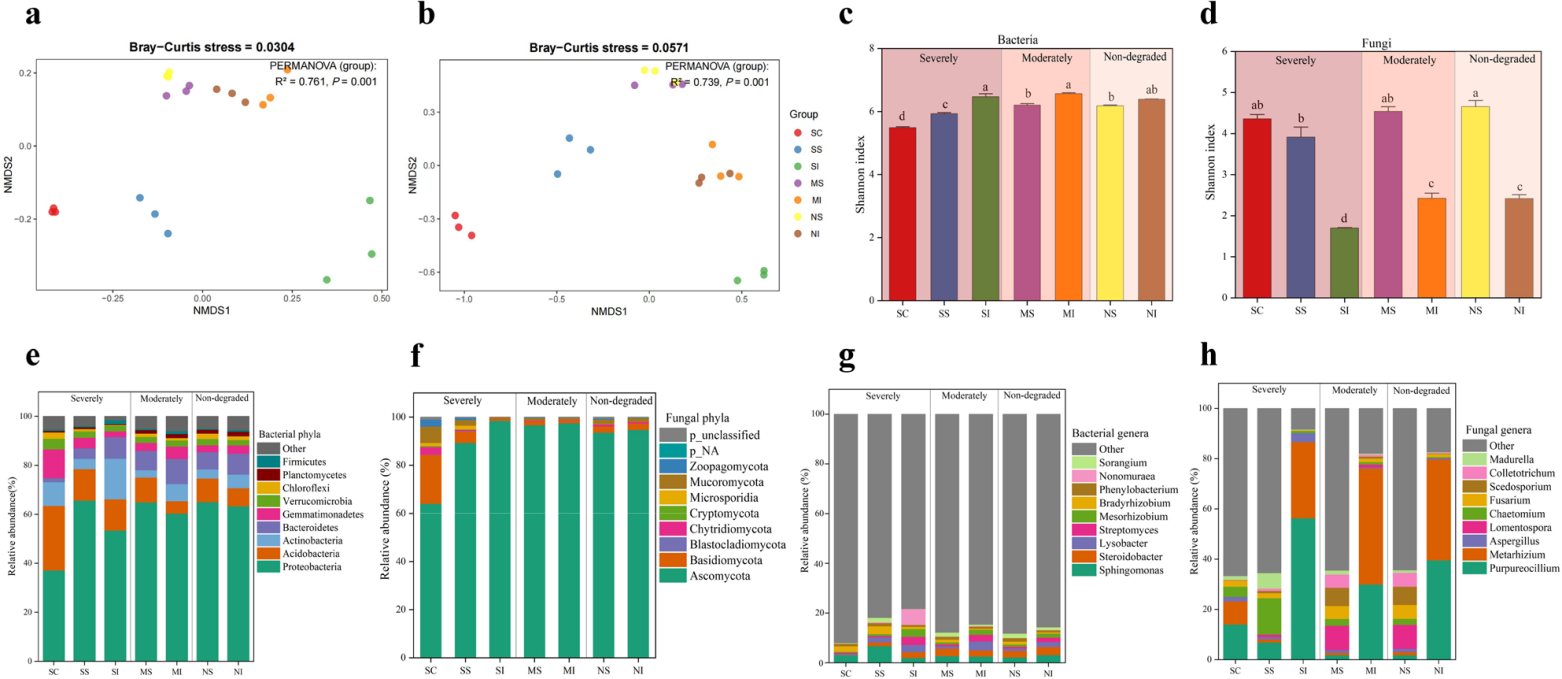
Microbial community structure and diversity across treatments.Non‑metric multidimensional scaling (NMDS) ordination based on Bray–Curtis distances for **a** bacterial and **b** fungal communities. The Shannon diversity index for **c** bacterial and **d** fungal communities (mean ± SE). Different lowercase letters denote significant differences (*P* < 0.05; one‑way ANOVA with Tukey’s HSD test). Stacked bar charts showing the relative abundance of the top 10 **e** bacterial phyla, **f** fungal phyla, **g** bacterial genera, and **h** fungal genera. In panel f, ‘p_unclassified’ denotes sequences unassigned at the phylum level, and ‘p_NA’ denotes low‑quality or unalignable sequences. SC, severely degraded soil, control; SS, severely degraded soil with straw addition; SI, severely degraded soil with straw combined with microbial inoculants; MS, moderately degraded soil with straw addition; MI, moderately degraded soil with straw combined with microbial inoculants; NS, non-degraded soil with straw addition; NI, non-degraded soil with straw combined with microbial inoculants

In line with the composition of the applied inoculant, treatments that combined straw with microbial inoculants showed significant greater relative abundance of *Bacillus* and *Metarhizium* compared to treatments with straw alone (Fig. 4a, b). Beyond the response of these specific taxa, the amendments exerted a comprehensive influence on the soil microbiome. NMDS analysis indicated distinct clustering of soil samples in ordination space, reflecting significant differences in bacterial and fungal community structures (*P* = 0.001; Fig. 5a, b). In terms of alpha diversity, the bacterial Shannon index increased in severely degraded soil under both amendments. In contrast, the fungal Shannon index was consistently lower in straw-inoculant treatments than in those with straw alone (Fig. 5c, d). At the phylum level, bacterial communities were dominated by *Proteobacteria*, *Actinobacteria*, *Acidobacteria*, and *Bacteroidetes*, with *Proteobacteria* being the most abundant phylum across all treatments (Fig. 5e). At the genus level, the relative abundances of *Lysobacter*, *Streptomyces*, and *Mesorhizobium* were notably higher in treatments that received both straw and microbial inoculants (SI, MI, NI) than in those receiving straw alone (SS, MS, NS) (Fig. 5g). In the fungal community, the phylum-level dominant taxa was *Ascomycota* (89.22%) (Fig. 5f). Compared to the control, both SS and SI treatments significantly increased the relative abundance of *Ascomycota* while markedly reducing that of *Basidiomycota*. At the genus level, *Purpureocillium* emerged as the predominant microflora across all seven treatments (Fig. 5h). Notably, under the combined application of straw and microbial inoculants, both *Purpureocillium* and *Metarhizium* (*Ascomycota*) were significantly enriched (*P* < 0.05).

#### Identification of differentially abundant taxa

**Fig. 6:**
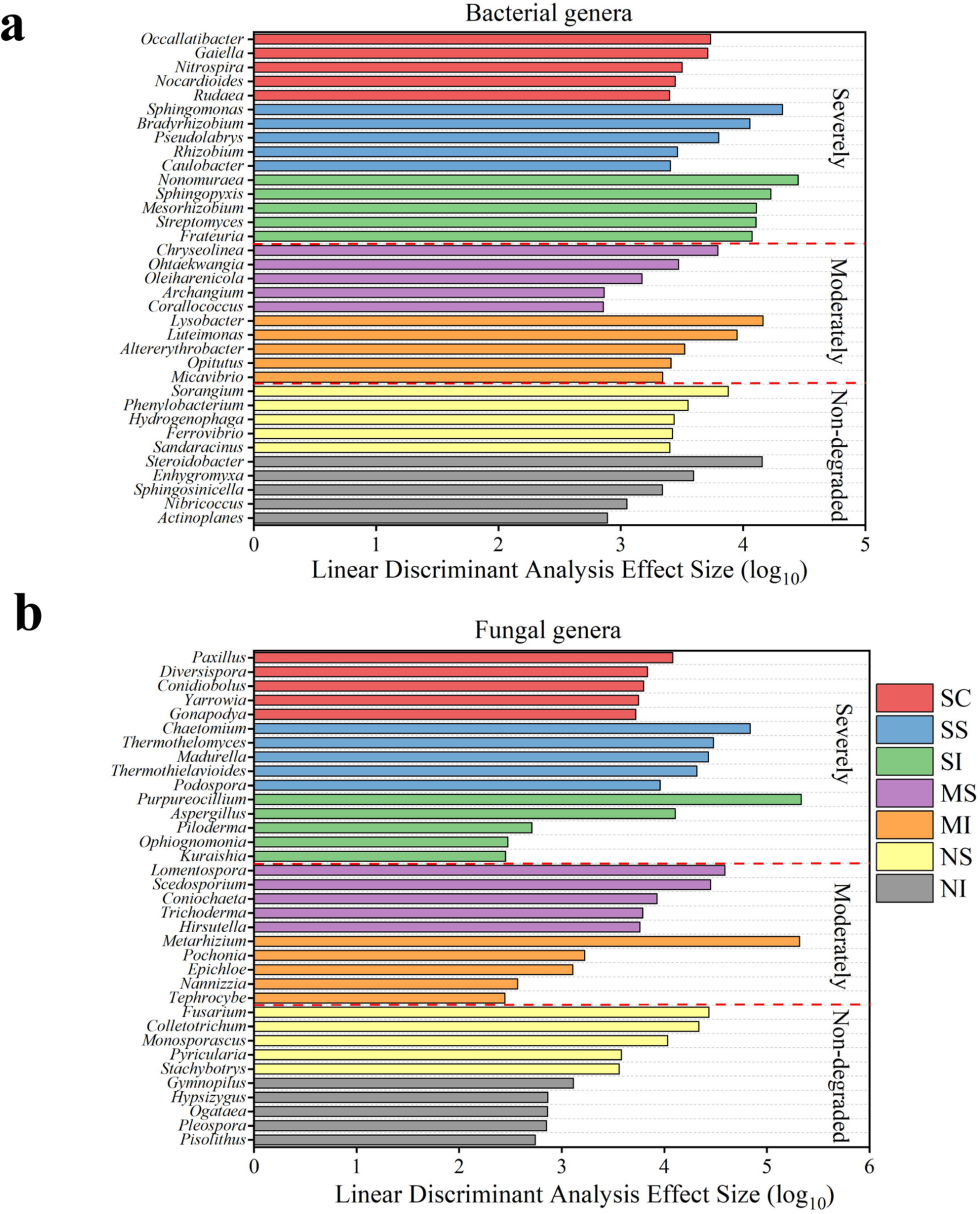
LEfSe analysis of microbes at the genus level across treatments. **a** Bacterial genera. **b** Fungal genera. The length of each bar represents the LDA score (Linear Discriminant Analysis Effect Size, log10-scaled), which estimates the effect size of each taxon in differentiating the treatment groups. Only taxa with an LDA score > 2.0 and a statistical significance of *P* < 0.05 (Kruskal-Wallis test) are shown. SC, severely degraded soil, control; SS, severely degraded soil with straw addition; SI, severely degraded soil with straw combined with microbial inoculants; MS, moderately degraded soil with straw addition; MI, moderately degraded soil with straw combined with microbial inoculants; NS, non-degraded soil with straw addition; NI, non-degraded soil with straw combined with microbial inoculants

LEfSe analysis identified a total of 1723 bacterial and 273 fungal genera that exhibited differential abundance in response to the amendments. The most pronounced bacterial responses were observed within the *Proteobacteria*. In severely degraded soils, the control treatment was characterized by *Occallatibacter* (*Acidobacteria*), while the straw and straw+inoculants treatments were distinguished by *Sphingomonas* (*Proteobacteria*) and *Nonomuraea* (*Actinobacteria*), respectively. In moderately degraded soils, *Chryseolinea* (*Bacteroidetes*) and *Lysobacter* (*Proteobacteria*) were indicative of the MS and MI treatments. In non-degraded soils, significant differences were driven by *Sorangium* and *Steroidobacter* (both *Proteobacteria*). Among fungi, *Ascomycota* contained the majority of differential taxa. In severely degraded soils, the indicative genera shifted from *Paxillus* (*Basidiomycota*) in the control treatment to *Chaetomium* in SS and *Purpureocillium* in SI. Notably, in moderately degraded soils, the inoculated genus *Metarhizium* was a key differential taxon for the MI treatment, alongside *Lomentospora* for MS. In non-degraded soils, *Fusarium* and *Gymnopilus* (*Basidiomycota*) were the primary differential genera for the NS and NI treatments, respectively (Fig. 6).

#### Soil C-cycle microorganisms and gene co-occurrence networks

**Fig. 7:**
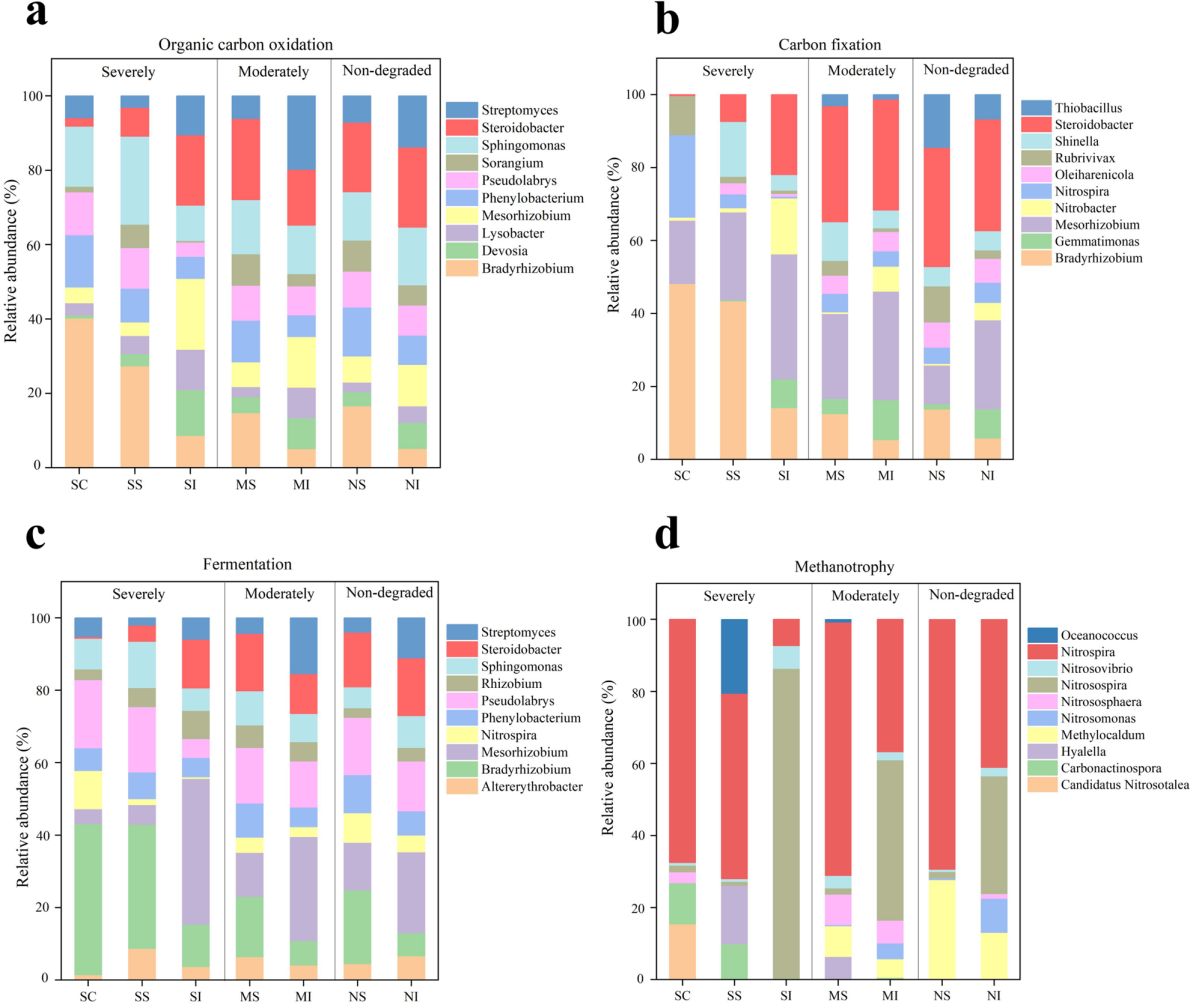
Contributions of soil microbial groups to soil C cycle functions at the genus level. **a** Organic carbon oxidation. **b** Carbon fixation. **c** Fermentation. **d** Methanotrophy. SC, severely degraded soil, control; SS, severely degraded soil with straw addition; SI, severely degraded soil with straw combined with microbial inoculants; MS, moderately degraded soil with straw addition; MI, moderately degraded soil with straw combined with microbial inoculants; NS, non-degraded soil with straw addition; NI, non-degraded soil with straw combined with microbial inoculants.

The functional contributions of major microbial genera to key soil C cycling processes were quantified based on metagenomic annotation (Fig. 7). *Bradyrhizobium* was the primary contributor to organic carbon oxidation, carbon fixation, and fermentation processes, as well as *Nitrospira* demonstrated higher diversity in the methanotrophy process. For organic carbon oxidation, the combined application of straw and microbial inoculants significantly reduced the relative abundance of *Bradyrhizobium* by 68.29%, 65.51%, and 68.99% in severely, moderately, and non-degraded soils, respectively, compared to straw application alone. Conversely, *Devosia* abundance increased substantially by 273.78%, 91.88%, and 85.94% under the same straw-inoculants treatments(Fig. 7a). Regarding carbon fixation, *Bradyrhizobium* exhibited a similar decreasing trend, while *Steroidobacter* abundance increased markedly from 0.32% in the control treatment to 30.66% in NI treatment (Fig. 7b). For fermentation processes, the straw-inoculants combination significantly enhanced *Mesorhizobium* abundance by 642.80%, 136.68%, and 69.17% in severely, moderately, and non-degraded soils, respectively, relative to straw-only treatments (Fig. 7c). In terms of methanotrophy, *Nitrosospira* was predominantly enriched in straw-inoculants treatments, accounting for 86.00%, 44.52%, and 32.66% of the communities in severely, moderately, and non-degraded soils, respectively (Fig. 7d).

**Fig. 8:**
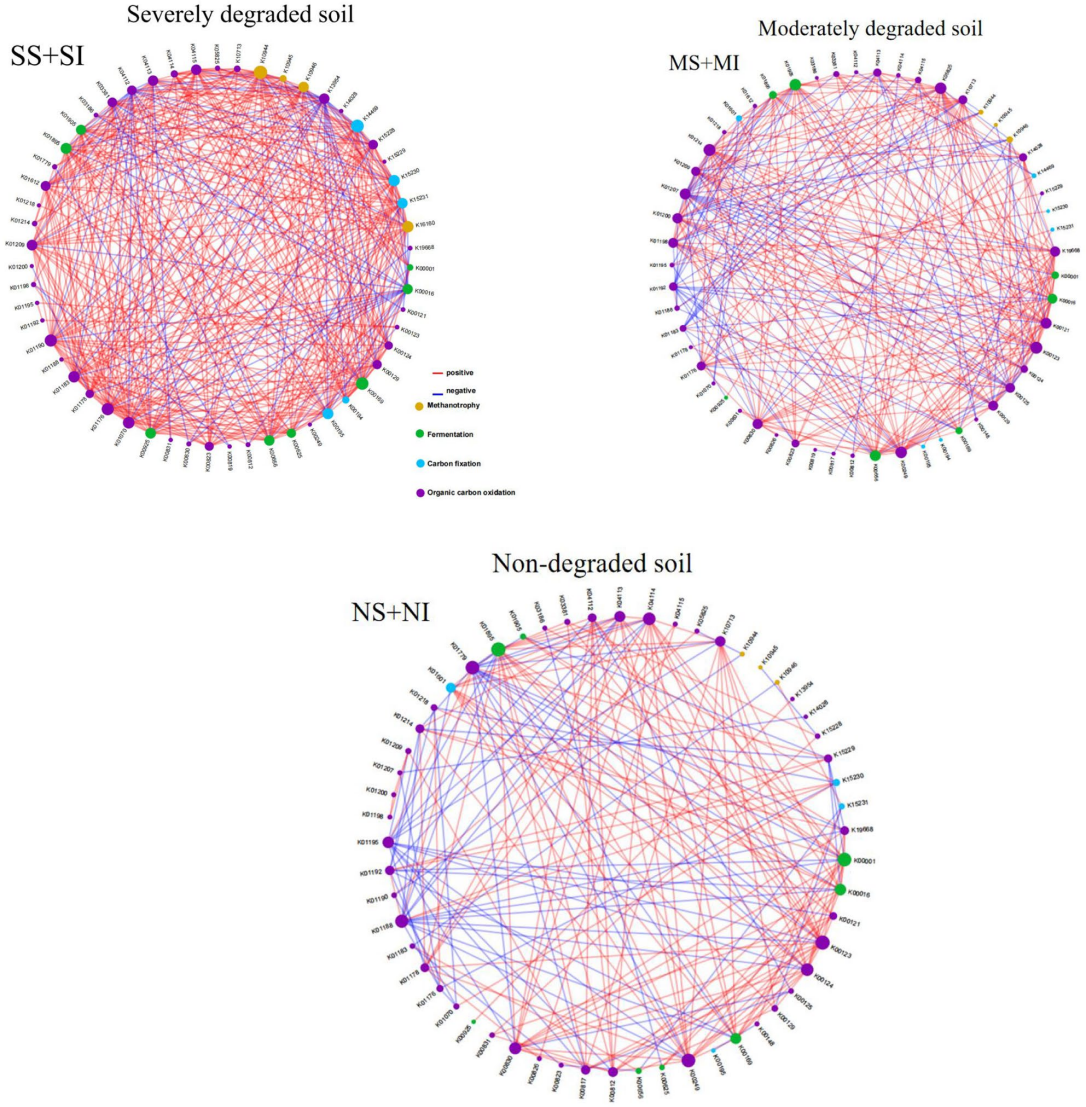
Occurrence network analysis showing of genes involved in soil C cycle under different degraded cinnamon soil. The node size shows the intensity of the links with other nodes (genes). Nodes with purple, bule, green and yellow colors represent the genes involved in organic carbon oxidation, carbon fixation, fermentation, and methanotrophy, respectively. SS, severely degraded soil with straw addition; SI, severely degraded soil with straw combined with microbial inoculants; MS, moderately degraded soil with straw addition; MI, moderately degraded soil with straw combined with microbial inoculants; NS, non-degraded soil with straw addition; NI, non-degraded soil with straw combined with microbial inoculants. The colored line shows the correlation of the two nodes, with the red line for the positive relationship and the blue line for the negative relationship. The basic topological properties of these networks are shown in Table S3

In moderately and non-degraded soils, the addition of straw combined with microbial inoculants significantly reduced the topological parameters (average degree and density) of the soil C cycle gene co-occurrence networks compared to severely degraded soil (Fig. 8, Table S3). Specifically, Straw combined with microbial inoculants addition decreased the node degree of genes involved in methanotrophy (e.g., K10945, K10946), fermentation (e.g., K00656, K00001, K01905), and carbon fixation (K15231), while increasing the proportion of negative correlations with genes for organic carbon oxidation (e.g., K01192, K01188).

A correlation analysis was performed between the abundance profiles of soil C cycle genes and soil physicochemical properties to determine the key environmental factors influencing microbial functional potential. Soil pH was significant determining factor controlling the structure of the C-cycling functional taxa (Fig. S2a). For example, soil pH was significant positively related to the abundances of the soil C genes coding for methanotrophy, fermentation and organic carbon oxidation (*P* < 0.05, *P* < 0.01, *P* < 0.05, respectively). Carbon fixation-related genes were strongly linked to SOC and AK (*P* < 0.05). Significant positive correlations were observed between several microbial taxa and C-cycling genes. Specifically, the abundances of *Actinobacteria*, *Gemmatimonadetes*, and *Chloroflexi* (bacteria), as well as *Basidiomycota* and *Mucoromycota* (fungi), were significantly correlated with range of C-cycling functional genes (Fig. S2b).

#### Potential microbial functions based on KEGG and CAZy analysis

##### Distinct microbial community

 structures under the straw and microbial inoculants treatments were associated with differentiated potential functional profiles across degradation levels. Likewise, it can be inferred from the metagenomic analysis that significant differences (*P* = 0.001) in microbial function based on the KEGG pathway and CAZyme were exhibited across all the soil samples (Fig. S3a-d). These differential pathways and modules were primarily enriched in categories of metabolism (e.g., oxidative phosphorylation, pyruvate metabolism, and purine metabolism), genetic information processing (e.g., ribosome), environmental sensing systems (e.g., quorum sensing, ABC transporters, and two-component system), and carbon utilization mechanisms (e.g., glycoside hydrolase, glycosyltransferase, and carbohydrate-binding module). Meanwhile, correlation analysis linked specific microbial genera to these functional potentials (Fig. S3e). Bacterial genera *Sphingomonas*, *Bradyrhizobium*, *Phenylobacterium* and *Sorangium* showed positive correlations with various community-level functions, while fungal genera *Chaetomium*, *Fusarium* and *Madurella* were strongly linked to core metabolic pathways including pyruvate and purine metabolism.

The distributions of key C-cycling genes differed among degradation levels (Fig. S4). In severely degraded soils, the abundance of the K01214 gene (related to starch degradation) was significantly higher in SS treatment than in SI (Fig. S4c). Conversely, in moderately degraded soils, the abundance of the K01207 gene (related to chitin degradation) was significantly higher in the straw-microbial inoculants treatment than in the straw-only treatment (Fig. S4d). Additionally, compared to the control, multiple functional genes were significantly enriched in both the SS and SI treatments (13 and 11 genes, respectively; Fig. S4a, b). These differentially abundant genes were predominantly associated with pathways for fatty acid degradation, oligosaccharide metabolism, histidine synthesis, and cellulase production.

## Discussion

### Degradation-level-specific structuring of microbial communities under amendment

In the present study, both straw application and straw combined with microbial inoculants increased bacterial alpha diversity, particularly in severely and moderately degraded soils (Fig. 5c). This aligns with the known role of organic inputs in enhancing bacterial diversity and fostering more complex communities [43, 44]. Such stabilized communities may further facilitate organic carbon decomposition and utilization [45]. This functional potential was reflected in the significant enrichment of copiotrophic *Proteobacteria* and cellulolytic *Ascomycota* with straw addition (Fig. 5e, f) [46, 47]. Meanwhile, microbial inoculants application significantly elevated the abundance of *Actinobacteria*, which are known for their robust metabolic and remediation capabilities, playing a vital role in organic matter turnover and C cycling [48]. However, compared to straw application alone, straw-microbial inoculants application markedly reduced fungal alpha diversity (Fig. 5d), consistent with the findings of previous studies [49, 50]. One plausible explanation for this discrepancy is that bacteria respond more rapidly to short-term perturbations, whereas fungal communities may require longer time frames to re-establish equilibrium, leading to an apparent decline in diversity [51]. This observation supports the notion that bacterial communities are highly sensitive indicators of soil degradation and play a pivotal role in mediating restoration processes [30].

The turnover of keystone taxa diverged according to the initial soil degradation level (Fig. 8). Consequently, identical amendments fostered distinct microbial assemblages and ecological outcomes in soils of different degradation states. In severely degraded soil, the community shifted from oligotrophic taxa characteristic of nutrient-poor environments (e.g., *Occallatibacter* within *Acidobacteria*) toward copiotrophic responders (Fig. 8a). Straw addition enriched *Proteobacteria* such as *Sphingomonas*, a genus frequently associated with disturbed or oligotrophic soils including degraded alpine wetlands, indicating a conserved response to resource limitation [52, 53]. In moderately degraded soil, the combined treatment enriched *Lysobacter* (*Proteobacteria*) and, most notably, *Metarhizium*. The enrichment of *Metarhizium* is notable, as it potentially links the inoculation to a specific functional niche (Figs. 4d and 5h), which aligns with the observed increase in corresponding functional genes (Fig. S4d). Particularly, the marked increase of *Metarhizium* may be attributed to its dual functionality in chitin degradation and biocontrol [54, 55]. This suggests a targeted enhancement of decomposition pathways for fungal-derived carbon (chitin) in these soils. In non-degraded soil, with higher inherent fertility and complex organic matter (Table S1), amendments favored taxa capable of degrading more recalcitrant compounds. For example, bacteria like *Steroidobacter*, known for sterol and lipid degradation, and fungi such as the ligninolytic *Gymnopilus* were enriched (Fig. 8) [56, 57]. This degradation-dependent shift underscores that the restorative amendments did not impose a uniform community but rather interacted with the pre-existing soil context.

### Degradation-level-specific regulation of carbon cycling gene networks

The response of key C cycling genes to amendments was strongly dependent on soil degradation level. Notably, in severely degraded soils, straw application alone showed significantly higher abundance of the K01214 gene (isoamylase) compared to straw-inoculants treatment (Fig. S4c). As a key gene in starch degradation (GH13 subfamily), K01214 hydrolyzes amylopectin to release linear oligosaccharides, synergizing with alpha-amylase (GH13) for complete starch breakdown [58]. The pronounced response of severely degraded soils to straw amendment can be attributed to labile carbon limitation, a key constraint on microbial activity in these soils [59]. This limitation predisposes the microbial community to a strong priming effect upon the input of readily degradable organic carbon, a response that is markedly more intense than in healthier soils [59, 60]. In contrast, moderately degraded soils showed a significant increase in the chitinase K01207 gene under straw-inoculant co-application (Fig. S4d), consistent with enriched *Metarhizium* fungi and elevated chitin degradation gene redundancy (Fig. 5h, Fig. S6b). Chitin degradation-derived N-acetylglucosamine provides *Metarhizium* a nutritional competitive advantage in nutrient-limited soils [61], and chitinolytic microbes further mediate microbial necromass C accumulation via glucosamine metabolism, a key process for soil C cycling [62].

Beyond the degradation-specific responses, the combined application of straw and microbial inoculants generally reduced the relative abundance of genes linked to methanotrophy (e.g., K10944, K10946) and several fermentation steps (e.g., K00656, ackA, adh), compared to straw addition alone (Figs. 2 and 3). This may be linked to the addition of inoculant decrease in soil pH (Fig. 1a), as these pathways show significantly positive correlations with pH (Fig. S2a). This suggests that the pH shift, likely driven by microbial metabolic activity, was an integral part of the environmental change associated with the functional gene reshaping [51, 63, 64]. Furthermore, both amendment treatments decreased the abundance of the *fdoG* gene (K00123), which encodes a key subunit of formate dehydrogenase involved in C1 metabolism [47]. Initially, straw input likely triggered a rapid increase in r-strategists and the expression of glycoside hydrolase (GH) genes, leading to a strong priming effect and high respiratory carbon loss—a scenario of low carbon use efficiency (CUE) [65, 66]. The subsequent down regulation of methanotrophic and fermentative genes, within the context of a modified pH environment, suggests a microbial shift away from metabolic strategies with lower energy yield or higher carbon loss potential (e.g., strict anaerobic fermentation), toward more efficient aerobic processes for substrate utilization [67].

Co-occurrence network analysis of C-cycling genes revealed a reshaping of functional interactions along the degradation gradient. Counter to the expectation, the most connected and complex gene network was sustained in the severely degraded soil, not in the non-degraded soil (Fig. 8, Table S3) [68]. This pattern suggests a shift in microbial ecological strategy driven by soil stress [69]. In severely degraded soil, this pattern aligns with the “Stress Gradient Hypothesis”, which predicts that increasing environmental stress (here, critically low SOC content as shown in Fig. 1b) shifts microbial interactions from competition to facilitation, resulting in a more connected and complex “stress-induced cooperative network” [64]. In contrast, non-degraded soils with sufficient resource support an “ecological niche-differentiated efficient network”, where functional genes are organized into specialized, semi-autonomous modules (e.g., chitin or lignin degradation) with dense intra-module but sparse inter-module connections, enhancing functional efficiency and stability [60, 70].

### Soil degradation status shapes microbiome functional response to amendment

Based on CAZymes annotation analyses, glycoside hydrolases (GHs) families, representing the key functional gene groups for decomposition (cellulose, chitin, and starch), exhibited the highest relative abundance in soil microbial communities (Fig. S5a). The applied microbial inoculant contained *Bacillus subtilis*, a genus well known for secreting various hydrolytic enzymes and recognized as versatile decomposers of complex carbohydrates [71, 72]. Following straw and microbial inoculants application, we observed significant increases in gene abundance of several critical GH family enzymes: alpha-glucosidase (GH31), beta-glucosidase (GH3), and alpha-amylase (GH13) (Fig. S5b). The high genetic potential for producing hydrolytic enzymes, reflected in the abundance of GH genes, may represent a genomic signature of r-strategists [73]. This genetic repertoire enables them to rapidly exploit available substrates, aligning with their high-growth lifestyle. The inoculated *B*. *subtilis*, as a classic r-strategist, likely played a key role in this functional shift, contributing to the observed enhancement of the community’s decomposition potential [74]. Importantly, consistent with the enrichment of carbon-utilization functions, the dominant enriched taxa (e.g., the positively correlated *Sphingomonas*, *Bradyrhizobium*) were found to harbor a high genetic potential for synthesizing corresponding key CAZymes (Fig. S3e), including glycosyl transferases and carbohydrate esterases. This enhanced enzymatic synthesis potential at the genetic level facilitated a more comprehensive breakdown of complex straw-derived organic matter [31], a finding consistent with shifts in microbial community composition observed in our previous metabolite analysis [12].

During the decomposition of soil organic matter, carbon-degrading genes can be categorized based on the complexity of their target compounds, from the most labile (e.g., starch) to the most recalcitrant (e.g., lignin) [75]. This hierarchy is determined by both substrate chemical structure and the degree of microbial synergy required for breakdown [47]. Critically, our results demonstrate that the application of microbial inoculants universally increased the functional redundancy of chitin-degrading genes, particularly in degraded soils (Fig. S6). This state-dependent selection is further corroborated by significant correlations between initial soil properties and the responsiveness of key functional genes (Fig. S7). The enrichment of starch-degrading genes in severely degraded soils, despite low overall fertility, reveals the dominance of carbon limitation as a selective force, channeling community function toward scavenging the most labile substrates. This nutrient-driven specialization aligns with ecological theory where resource scarcity favors taxa with high-affinity, rapid-uptake strategies [76].

This targeted enhancement stands in stark contrast to the inherently stable functional redundancy observed in non-degraded soils, which showed minimal response to inoculation (Fig. S6c). It has been documented that *Bacillus* species are prevalent and functionally important in chitin-degrading microbial communities, responsible for roughly 20–30% of the chitinolytic enzyme activity [74, 77]. Since severely and moderately degraded soils exhibit initial deficiencies in chitinolytic microorganisms, inoculation with *Bacillus*-containing microbial inoculants significantly enhanced soil chitinase GH19 activity through successful colonization (Fig. S6a, b). This phenomenon demonstrates how soil degradation alters microbial ecological strategies, while the inherent stability of native microbial communities in non-degraded soils renders them less responsive to inoculant interventions [78, 79]. This underscores that the efficacy of microbial amendments is not intrinsic to the inoculant itself, but is contingent upon the pre-existing functional state of the soil microbiome.

Based on these findings, we propose a diagnostic-driven framework for restoring degraded cinnamon soils. The initial step involves assessing the soil’s functional state using key indicators such as SOC content and pH [63, 80]. For soils diagnosed as degraded, a two-step strategy is recommended: apply straw to alleviate carbon limitation, followed by the application of a tailored microbial inoculant. Specifically, for severely and moderately degraded soils, we propose applying a composite inoculant combining chitinolytic specialists with high-efficiency cellulolytic bacteria concurrently with straw at a field-applicable ratio (e.g., 1–5% of straw mass) to synergistically enhance the processing of complex organic matter and promote carbon sequestration. In contrast, for non-degraded soils where inoculant effects are marginal, priority should be given to optimizing agronomic practices, such as precision fertilization, to maintain the health and stability of the resident microbiome, rather than large-scale microbial inoculation. This framework translates our mechanistic insights into an actionable tool, advancing soil restoration from blanket applications toward precision practices based on functional diagnosis.

## Conclusions

This study elucidates the mechanisms by which straw and microbial inoculants restore degraded cinnamon soils through the regulation of microbial communities and carbon cycling genes. Our findings demonstrate that straw and straw-microbial inoculants significantly improved soil properties– enhancing AN and MBC in severely degraded soils, increasing SOC in moderately degraded soils, and boosting available nutrients in non-degraded soils, with soil pH identified as a key driver of C cycling functional community structure. Straw-microbial inoculants applications increased bacterial alpha diversity but reduced fungal diversity, and notably enhanced the complexity of C cycling gene networks in severely degraded soils. Functionally, specific bacterial genera (e.g., *Sphingomonas*, *Bradyrhizobium*) were linked to transport and hydrolysis functions, whereas fungal genera (e.g., *Chaetomium*, *Fusarium*) were associated with energy metabolism pathways. At the level of catabolic genes, straw addition significantly increased the abundance of the amylase gene K01214 (encoding α-amylase for starch hydrolysis) in severely degraded soils, whereas straw-inoculants combination enhanced the chitinase gene K01207 (encoding chitinase for chitin hydrolysis) abundance in moderately degraded soils. Consequently, we suggest for precision interventions based on soil functional diagnosis: applying targeted straw inputs and tailored microbial inoculants (e.g., chitinolytic-cellulolytic SynCom at 1–5% of straw mass) to rebuild microbial functional potential in degraded soils, and adopting optimized agronomic management practices (e.g., precision fertilization) to preserve microbiome stability in non-degraded soils. To further bridge genetic potential with ecosystem function, future research should integrate direct measures of microbial activity, such as extracellular enzyme assays and metatranscriptomic (mRNA) sequencing, with metagenomic surveys. This will be crucial to confirm the expression and in situ activity of the identified functional genes and to fully elucidate the metabolic pathways driving carbon turnover in restored soils.

## Electronic Supplementary Material

Below is the link to the electronic supplementary material.

Supplementary Material 1

Supplementary Material 2

Supplementary Material 3

Supplementary Material 4

## Data Availability

The DNA assembly data generated in this study have been deposited in the OMIX repository at the China National Center for Bioinformation (CNCB) under accession number OMIX012942. Soil physicochemical property data are provided in the manuscript as Additional file (1) The OTU-level abundance table, taxonomic annotations, and microbial diversity metrics are provided as Additional file (2) The carbon cycling gene abundance table and taxonomic characteristics are provided as Additional file 3.

## Abbreviations

SOC: Soil organic carbon
C: Carbon
TOC: Total organic carbon
SOM: Soil organic matter
AN: Alkali-hydrolyzable nitrogen
AK: Available potassium
AP: Available phosphorus
MBC: Microbial biomass carbon
KEGG: Kyoto Encyclopedia of Genes and Genomes
CAZyme: Carbohydrate-Active Enzyme
NMDS: Non-metric multidimensional scaling
PERMANOVA: Permutational multivariate analysis of variance
LEfSe: Linear discriminant analysis effect size
LDA: Linear Discriminant Analysis
STAMP: Statistical Analysis of Metagenomic Profiles
CUE: Carbon use efficiency
AA: Auxiliary activities
CBM: Carbohydrate-binding modules
CE: Carbohydrate esterases
GH: Glycoside hydrolases
GT: Glycosyl transferase
PL: Polysaccharide lyases
SynCom: Synthetic microbial community
