# Supplementary material for: Microbial inoculants with straw mediate degradation-level-specific changes in soil carbon cycling genes and microbial community
Source: Environ Microbiome. 2026 May 4;21:82. doi: 10.1186/s40793-026-00898-4 (PMC13317363; doi:10.1186/s40793-026-00898-4)
Supplement: Supplementary file 4 — Supplementary Material 4 [file 40793_2026_898_MOESM4_ESM.docx]

**Supplementary materials**

**Microbial inoculants with straw mediate degradation-level-specific changes in soil carbon cycling genes and microbial community**

Yu Han^[[1]](#footnote-0)^, Jiaxin Cui, Xiantong Huang, Ping Guo^*^, Shiqi Yang^[[2]](#footnote-1)^ *

Institute of Environment and Sustainable Development in Agriculture, Chinese Academy of Agricultural Sciences, Beijing, 100081, China

* Corresponding author:

Ping Guo, E-mail: guoping@caas.cn (P. Guo)

Institute of Environment and Sustainable Development in Agriculture, Chinese Academy of Agricultural Sciences, Beijing, 100081, China.

Shiqi Yang, E-mail: shiqiyang@126.com (S. Yang)

Institute of Environment and Sustainable Development in Agriculture, Chinese Academy of Agricultural Sciences, Beijing, 100081, China.

**Appendix S1: Detailed protocols for inoculant quality control and pot experiment management**

**S1.1 Quality control of the composite microbial inoculant**

Prior to application, the composite microbial inoculant powder (comprising *Metarhizium* *anisopliae* spore powder and *Bacillus* *subtilis* powder, supplied by Ningxia Zhongwei Tech-Biotech Co., Ltd.) was subjected to quality control verification.

*Metarhizium* *anisopliae* spore quantification: Viable spore concentration was determined in our laboratory by serial dilution plating. Briefly, the spore powder was serially diluted in sterile water, and 0.1 mL aliquots of an appropriate dilution were spread on Potato Sucrose Agar with Yeast Extract (PSAY) plates. After incubation at 25°C for 3–5 days, colonies were counted to calculate the concentration, which was confirmed to be 1 × 10¹⁰ viable spores g⁻¹.

*Bacillus* *subtilis* quality assurance: According to the manufacturer's specifications, the *B*. *subtilis* component was produced via liquid fermentation, resulting in a broth with a viable cell concentration of ≥1×10¹⁰ CFU mL⁻¹ and a spore formation rate exceeding 90%. The subsequent drying and powdering process was designed to maintain this high viability.

The two component powders were pre-mixed by the supplier at a fixed, proprietary ratio to ensure functional synergy and batch-to-batch consistency for all pot experiments.

**S1.2 Preparation and application of amendments**

For treatments requiring the microbial inoculant, a calculated mass of the composite inoculant powder (equivalent to 5% of the soil dry mass) was first uniformly blended with the corresponding amount of crushed maize straw (also 5% soil mass, cut to 1 cm in length). This straw-inoculant mixture was then thoroughly incorporated into the bulk soil (5 kg per pot) to ensure homogeneous distribution of the amendments. Based on the verified *Metarhizium* concentration and the 5% (w/w) application rate, the target introduction of *Metarhizium* spores into the soil was 5 × 10⁸ spores g⁻¹ dry soil. The *Bacillus* component was co-introduced at a proportionally equivalent rate according to the fixed formulation ratio.

**S1.3 Sowing management to mitigate decomposition heat**

The pots were randomly put in a greenhouse under natural sunlight at room temperature and managed as previously described [1]. Since straw decomposition releases heat, alfalfa (*Medicago sativa* L. cv. WL358HQ) was sown only after the straw decomposition process stabilized to prevent seedling damage from excessive temperatures. The decomposition process exhibited distinct stages, and when the temperature of the middle soil layer (13 cm below the pot rim) stabilized at ambient temperature (26 ± 1°C), it indicated that the straw had entered the mature decomposition phase, allowing subsequent sowing. After seedling emergence, growth was monitored and thinned as needed. No additional fertilization was applied during alfalfa growth, and weeds were regularly removed to ensure proper water management.


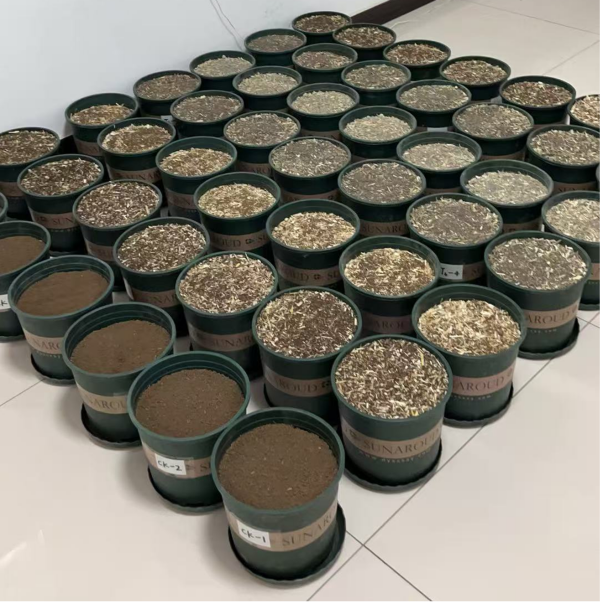


**Fig. S1 Layout of the pot experiment.** The photograph shows the seven treatment pots arranged sequentially from left to right. Treatments, applied to soils of three degradation levels, are as follows: SC (severely degraded control), SS (severely degraded with straw), SI (severely degraded with straw and inoculant), MS (moderately degraded with straw), MI (moderately degraded with straw and inoculant), NS (non-degraded with straw), and NI (non-degraded with straw and inoculant). The photo was taken prior to alfalfa sowing.

**Appendix S2: Description of soil physical and chemical determination**

Soil pH was determined using the water immersion potentiometric method (soil/water ratio of 1:2.5) [2]. Soil organic carbon (SOC) was determined using the potassium dichromate volumetric method, which involves oxidation of organic matter with potassium dichromate (K_2_Cr_2_O_7_) and sulfuric acid (H_2_SO_4_) [3]. Alkali-hydrolyzable nitrogen (AN) was measured by the alkaline hydrolysis diffusion method, where the ammonia released during hydrolysis is captured in a boric acid solution and titrated with a standard acid solution[4]. Available potassium (AK) was assessed using 1 mol/L NH_4_OAc extraction followed by flame photometry, and available phosphorus (AP) was determined using the 0.5 mol/L NaHCO_3_ extraction method [5, 6]. Microbial biomass carbon (MBC) was measured by the chloroform fumigation-extraction method, where the carbon released from lysed microbial cells after chloroform fumigation is extracted with potassium sulfate (0.5 M K_2_SO_4_) and quantified by a total organic carbon analyzer, with the difference in extractable carbon between fumigated and non-fumigated soil samples calculated as MBC [7].

**Appendix S3: Description of soil DNA extraction and metagenome sequencing**

DNA was extracted from soil subsamples (0.25 g, stored at -80 °C) using the E.Z.N.A. Soil DNA Kit (Omega Bio-tek, Inc., USA) following the manufacturer's instructions. The ﬁnal DNA concentration and quality of the genomic DNA were checked by NanoDrop 2000 spectrophotometer (Thermo Scientific Inc., USA). The qualified DNA samples were then sent to Allwegene Company (Beijing, China) for library preparation and shotgun metagenomics sequencing. Sequencing of the metagenomic DNA shotgun was performed using the Illumina HiSeq 2500 platform (Illumina, Inc., San Diego, CA, USA). For this, ∼5.0 Gbp paired-end reads (150 bp in length) were produced after contaminant and quality filtering. The cleaned sequences were de novo assembled into contigs using the MEGAHIT assembler [8]. The selected contigs were uploaded to and annotated by the Integrated Microbial Genomes pipeline to derive abundance tables for genes based on the Kyoto Encyclopedia of Genes and Genomes (KEGG) database [9]. The functional annotation and taxonomic assignment of the sequences obtained for each sample were performed, and then 61 functional genes involved in soil C cycle were selected. They were classified into four categories according to their functions in the soil C cycles. The KO numbers, gene names, functions, and classifications of the genes associated with soil C cycling are shown in Table S2. Additionally, to identify the functional characteristics of the microbial communities, metagenomic reads were annotated by comparison them with the KEGG database, and the contribution percentages of microbial genus to these processes were calculated to assess the functional contributions of major microbial groups to soil C cycling at the community level.

Carbohydrate-Active Enzyme (CAZyme) annotation was performed by comparing the amino acid sequences of the non-redundant gene set to the CAZy database using hmmscan from the HMMER (v3.3) software package [10]. The abundance of each CAZyme was subsequently calculated as the sum of the abundances of all genes encoding that enzyme [11]. Annotation against the KEGG and CAZy databases provides insights into functional and species contributions, and functional gene abundance, respectively.

**References**

1. Han Y, Cui J, Guo P, Yang S. Remediation effects of straw combined with microbial agents on cinnamon soils with varying degradation based on metagenomics and untargeted metabolome. Environ Res 2025; 285:15. https://doi.org/10.1016/j.envres.2025.122649.
2. Zeng J, Liu X, Song L, Lin X, Zhang H, Shen C, et al. Nitrogen fertilization directly affects soil bacterial diversity and indirectly affects bacterial community composition. Soil Biol Biochem 2016; 92:41-49. https://doi.org/10.1016/j.soilbio.2015.09.018.
3. Fu Y, Xu Y, Wang Q, Van Zwieten L, Liang C, Xu J, et al. Deciphering the microbial players driving straw decomposition and accumulation in soil components of particulate and mineral-associated organic matter. Soil Biol Biochem 2025; 209:12. https://doi.org/10.1016/j.soilbio.2025.109871.
4. Xie HT, Yang XM, Drury CF, Yang JY, Zhang XD. Predicting soil organic carbon and total nitrogen using mid - and near-infrared spectra for Brookston clay loam soil in Southwestern Ontario, Canada. Can J Soil Sci 2011; 91:53-63. https://doi.org/10.4141/CJSS10029.
5. Islam A, Karim AJMS, Solaiman ARM, Islam MS, Salequea MA. Eight-year long potassium fertilization effects on quantity / intensity relationship of soil potassium under double rice cropping. Soil Till Res 2017; 169:99-117. https://doi.org/10.1016/j.still.2017.02.002.
6. Xiong W, Zhao Q, Zhao J, Xun W, Li R, Zhang R, et al. Different continuous cropping spans significantly affect microbial community membership and structure in a vanilla-grown soil as revealed by deep pyrosequencing. Microb Ecol 2015; 70:209-18. https://doi.org/10.1007/s00248-014-0516-0.
7. Vance ED, Brookes PC, Jenkinson DS. An extraction method for measuring soil microbial biomass C. Soil Biol Biochem 1987; 19:703-7. <https://doi.org/10.1016/0038-0717(87)90052-6.>
8. Li D, Liu CM, Luo R, Sadakane K, Lam TW. MEGAHIT: an ultra-fast single-node solution for large and complex metagenomics assembly via succinct de Bruijn graph. Bioinformatics 2015; 31:1674-1676. <https://doi.org/10.1093/bioinformatics/btv033.>
9. Kanehisa M, Goto S. KEGG: kyoto encyclopedia of genes and genomes. Nucleic Acids Res 2000; 28:27-30. <https://doi.org/10.1093/nar/28.1.27.>
10. Eddy SR. Accelerated profile HMM searches. PLoS Comput Biol 2011; 7:16. https://doi.org/10.1371/journal.pcbi.1002195.
11. Lombard V, Ramulu HG, Drula E, Coutinho PM, Henrissat B. The carbohydrate-active enzymes database (CAZy) in 2013. Nucleic Acids Res 2014; 42:D490-495. https://doi.org/10.1093/nar/gkt1178.

**Supplementary Tables**

**Table S1** **Basic soil physicochemical parameters of cinnamon soils under different degradation levels preceding straw and microbial inoculants application.**

| Group | pH | SOC  (mg/kg) | AK  (g/kg) | AP  (mg/kg) | AN  (mg/kg) | MBC  (mg/kg) |
| --- | --- | --- | --- | --- | --- | --- |
| Severely degraded soil | 5.91±0.03a | 13.47±0.27a | 0.17±0.03a | 16.92±0.61a | 85.63±3.29a | 61.61±5.65a |
| Moderately degraded soil | 6.64±0.05b | 31.53±0.82b | 0.86±0.07b | 70.36±1.28b | 135.63±2.25b | 117.42±12.33b |
| Non-degraded soil | 7.14±0.03c | 103.13±1.20c | 1.72±0.06c | 127.21±2.40c | 217.81±2.82c | 361.63±20.75c |

Note: Sample data are presented as mean±SE, n=3. Different lowercase letters indicate significant differences (one-way ANOVA with Tukey’s HSD test, *P* < 0.05). SOC, soil organic carbon; AK, available potassium; AP, available phosphorus; AN, alkali-hydrolyzable nitrogen; MBC, microbial biomass carbon.

**Table S2** **The KO number, function descriptions, gene name and classification of the investigated genes related to soil C cycle.**

| Classification | KO number | Details for gene function | Corresponding gene |
| --- | --- | --- | --- |
| Genes involved in  methanotrophy | K10944 | Methane oxidation - Partculate methane monooxygenase | *pmoA* |
|  | K10945 | Methane oxidation - Partculate methane monooxygenase | *pmoB* |
|  | K10946 | Methane oxidation - Partculate methane monooxygenase | *pmoC* |
|  | K16160 | Methane oxidation - Soluble methane monoxygenase | *mmoB* |
| Genes involved in fermentation | K01905 | Acetogenesis | *acdA* |
|  | K00925 | Acetogenesis | *ack* |
|  | K01895 | Acetate to acetyl-CoA | *acs* |
|  | K00001 | Alcohol utilization | *adh* |
|  | K00016 | Lactate utilization | *ldh* |
|  | K00656 | Pyruvate <=> acetyl-CoA + formate | *pflD* |
|  | K00169 | Pyruvate oxidation | *porA* |
|  | K00625 | Acetogenesis | *pta* |
| Genes involved in carbon fixation | K01601 | CBB cycle - Rubisco | *Form II* |
|  | K14469 | 3 Hydroxypropionate cycle | *K14469* |
|  | K15230 | Reverse TCA cycle | *aclA* |
|  | K15231 | Reverse TCA cycle | *aclB* |
|  | K00194 | Wood Ljungdahl pathway | *cdhD* |
|  | K00195 | Wood Ljungdahl pathway | *cdhE* |
| Genes involved in organic carbon oxidation | K00823 | 4-aminobutyrate aminotransferase and related aminotransferases | *4-aminobutyrate aminotransferase and related aminotransferases* |
|  | K13954 | Acetaldehyde => Ethanol | *acetaldehyde => ethanol* |
|  | K00129 | Acetate => Acetaldehyde | *acetate => acetaldehyde* |
|  | K00249 | Fatty acid degradation | *acyl-CoA dehydrogenase* |
|  | K01176 | Amylolytic enzymes | *alpha-amylase* |
|  | K05825 | Aminotransferase class I and II | *aminotransferase class I and II* |
|  | K01209 | Hemicullulose debranching | *arabinosidase* |
|  | K00812 | Metabolism of organic sulfur \| Cysteine <-> 3-mercaptopyruvate | *aspB* |
|  | K04114 | Benzoyl-CoA reduction | *bcrA* |
|  | K04113 | Benzoyl-CoA reduction | *bcrB* |
|  | K04112 | Benzoyl-CoA reduction | *bcrC* |
|  | K04115 | Benzoyl-CoA reduction | *bcrD* |
|  | K01190 | Other oligosaccharide degrading | *beta-galactosidase* |
|  | K01188 | Cellulose degrading | *beta-glucosidase* |
|  | K01195 | Hemicullulose debranching | *beta-glucuronidase* |
|  | K01192 | Other oligosaccharide degrading | *beta-mannosidase* |
|  | K01198 | Other oligosaccharide degrading | *beta-xylosidase* |
|  | K00826 | Branched-chain amino acid aminotransferase/4-amino-4-deoxychorismate lyase | *branched-chain amino acid aminotransferase/4-amino-4-deoxychorismate lyase* |
|  | K01612 | Phenol => Benzoyl-CoA | *bsdC* |
|  | K03381 | Protocatechuate/Catechol degradation | *catA* |
|  | K19668 | Cellulose degrading | *cellobiosidase* |
|  | K01779 | Cellulose degrading | *cellulase* |
|  | K01183 | Chitin degrading | *chitiniase* |
|  | K10713 | Formaldehyde oxidation | *fae* |
|  | K00148 | Formaldehyde oxidation | *fdhA* |
|  | K00125 | Formate oxidation | *fdhB* |
|  | K00123 | Formate oxidation | *fdoG* |
|  | K00124 | Formate oxidation | *fdoH* |
|  | K01070 | Formaldehyde oxidation | *fghA* |
|  | K00121 | Formaldehyde oxidation | *frmA* |
|  | K01178 | Amylolytic enzymes | *glucoamylase* |
|  | K01207 | Chitin degrading | *hexosaminidase* |
|  | K00817 | Histidinol-phosphate/aromatic aminotransferase | *histidinol-phosphate/aromatic aminotransferase* |
|  | K01214 | Amylolytic enzymes | *isoamylase* |
|  | K01218 | Endohemicellulases | *mannan endo-1,4-beta-mannosidase* |
|  | K15228 | Methyl amine -> formaldehyde | *mauA* |
|  | K15229 | Methyl amine -> formaldehyde | *mauB* |
|  | K14028 | Methanol oxidation | *mxaF* |
|  | K00819 | Ornithine/acetylornithine aminotransferase | *ornithine/acetylornithine aminotransferase* |
|  | K00831 | Phosphoserine aminotransferase | *phosphoserine aminotransferase* |
|  | K01200 | Amylolytic enzymes | *pullulanase* |
|  | K00830 | Serine-pyruvate aminotransferase/archaeal aspartate aminotransferase | *serine-pyruvate aminotransferase/archaeal aspartate aminotransferase* |
|  | K03186 | Phenol => Benzoyl-CoA | *ubiX* |

**Table S3** **The properties of gene occurrence networks with the different degree of degradation soils.**

| Network properties | Severely degraded soil | Moderately degraded soil | Non-degraded soil |
| --- | --- | --- | --- |
| Nodes | 55 | 55 | 56 |
| Edges | 408 | 289 | 203 |
| Positive correlation | 336 | 204 | 130 |
| Negative correlation | 72 | 85 | 73 |
| Average degree | 14.84 | 10.51 | 7.25 |
| Diameter | 6.16 | 6.97 | 7.83 |
| Density | 0.27 | 0.19 | 0.13 |
| Clustering coefficient | 0.76 | 0.66 | 0.59 |
| Modularity | 0.22 | 0.24 | 0.34 |

**Supplementary Figures**

**a**


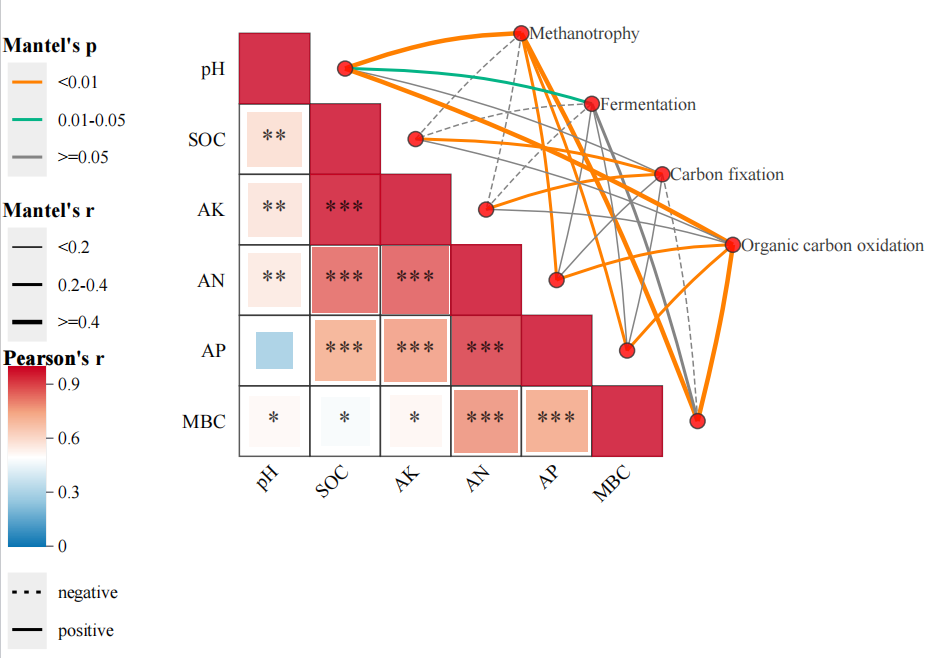
**b**


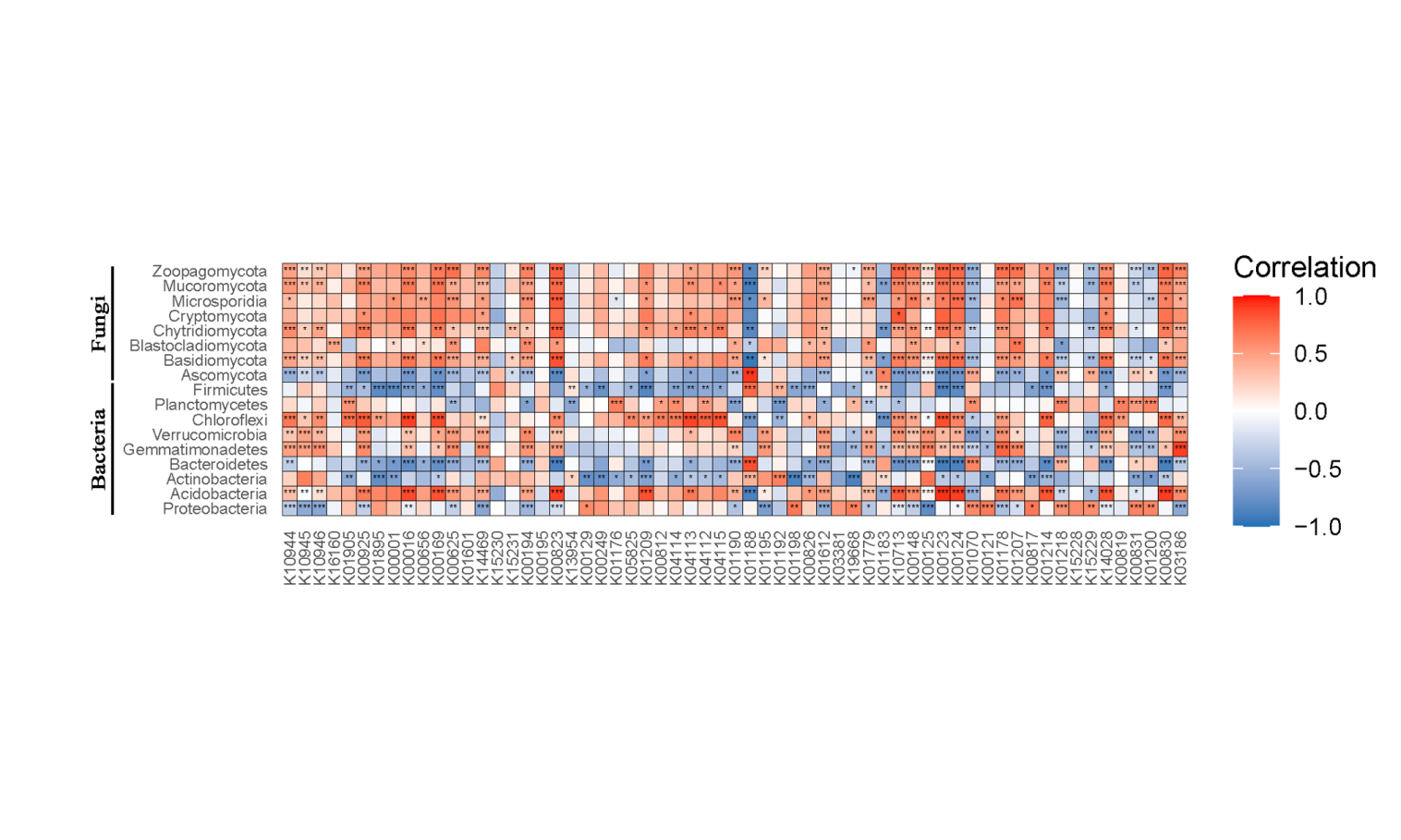


**Fig. S2 Environmental drivers of soil C cycle genes and gene compositions.** (a) Mantel test analysis of environmental factors with soil C-cycling functions, with a color gradient denoting Pearson's correlation coefficients. The solid and dashed lines indicate positive and negative correlations, respectively. Edge width corresponds to Mantel's r statistic for the corresponding distance correlations, and edge color denotes the statistical significance based on 999 permutations. (b) The relationship between major soil microbial phyla taxa (bacteria and fungi) and the relative abundance of soil C-cycling genes. *, *P* < 0.05; **, *P* < 0.01, ***, *P* < 0.001.

**a** **b**


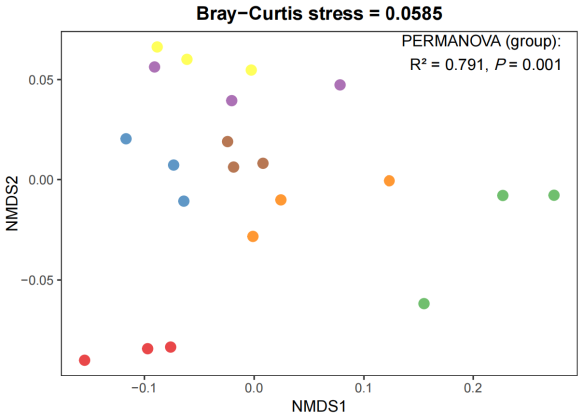

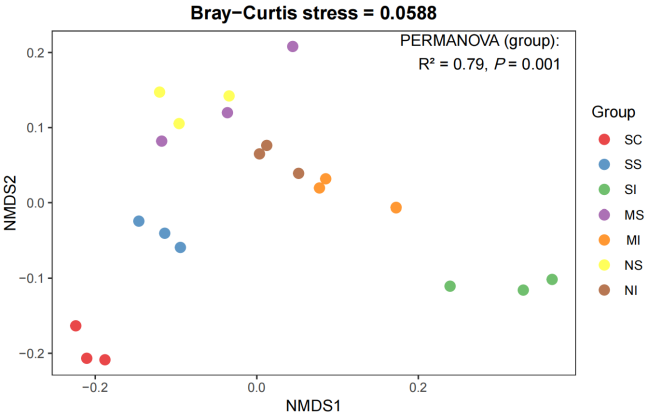


**c** **d**


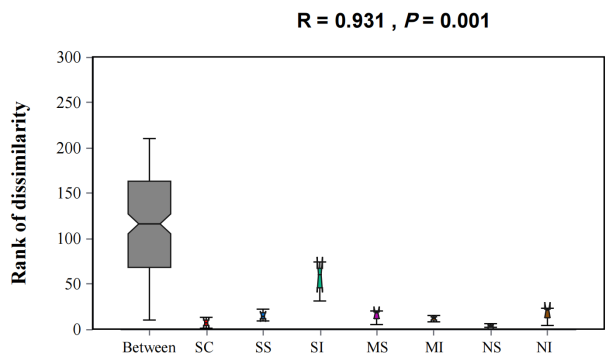

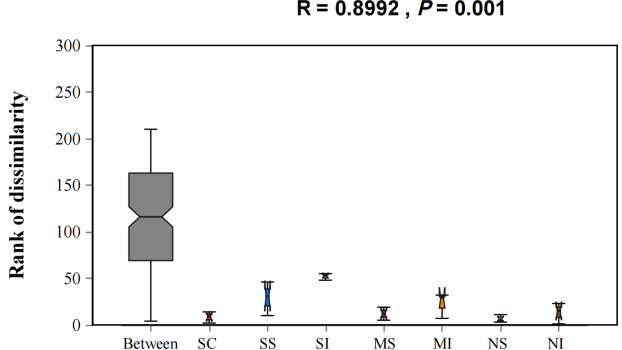


**e**


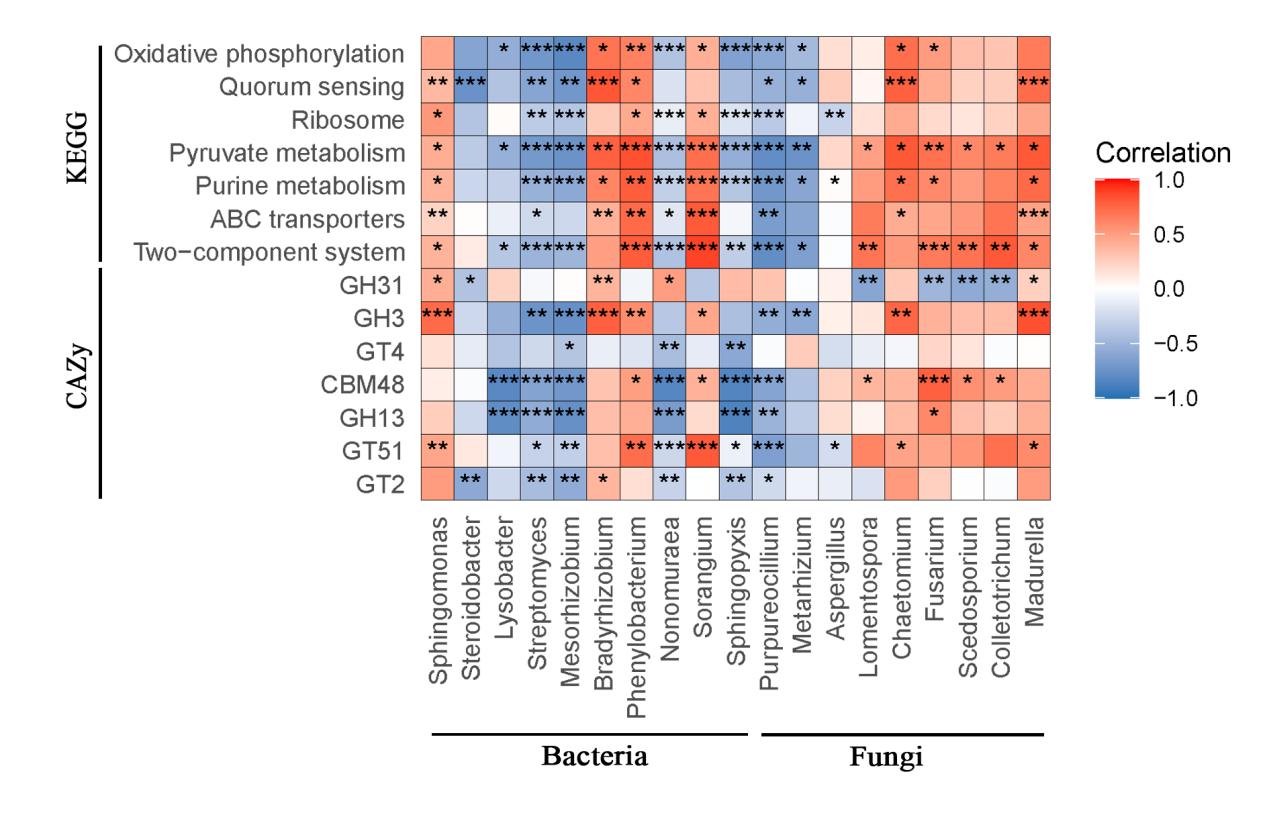


**Fig. S3 Differentiation of microbial functional profiles across treatments.** Non‑metric multidimensional scaling (NMDS) ordination based on Bray–Curtis distances showing the separation of microbial functional profiles according to (a) KEGG pathways (level 3) and (b) CAZy families. Analysis of similarities (ANOSIM) evaluating the statistical significance of differences among treatments for (c) KEGG pathways and (d) CAZy families. (e) Heatmap depicting the contribution of the major soil microbial taxa (bacterial and fungal genera) to the abundance of the top 7 KEGG pathways (level 3) and the top 7 CAZy families. *, *P* < 0.05; **, *P* < 0.01, ***, *P* < 0.001. CAZy family abbreviations: GH, glycoside hydrolases; GT, glycosyl transferases; CBM, carbohydrate‑binding modules. SC, severely degraded soil, control; SS, severely degraded soil with straw addition; SI, severely degraded soil with straw combined with microbial inoculants; MS, moderately degraded soil with straw addition; MI, moderately degraded soil with straw combined with microbial inoculants; NS, non-degraded soil with straw addition; NI, non-degraded soil with straw combined with microbial inoculants.

**a** **b**


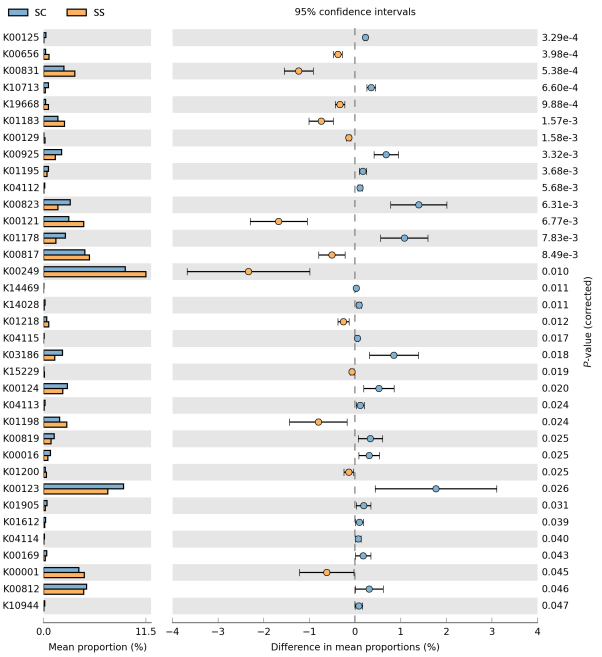

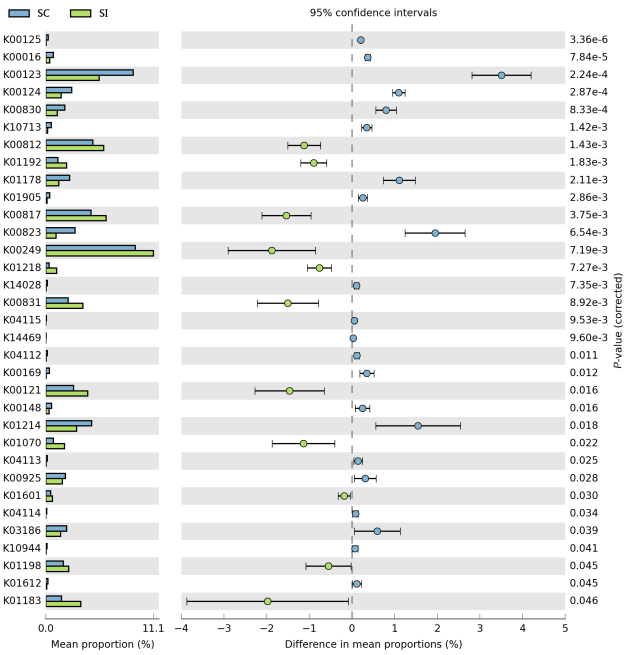


**c** **d**


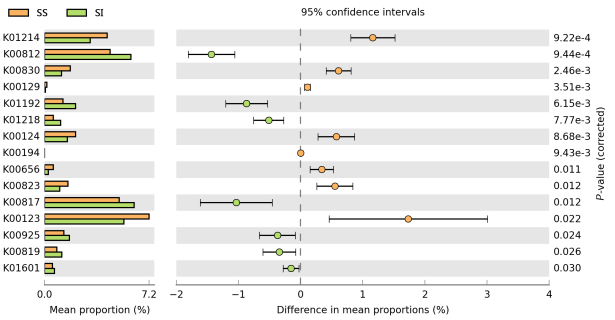

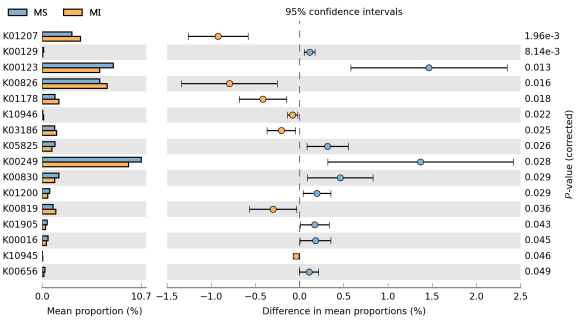


**e**


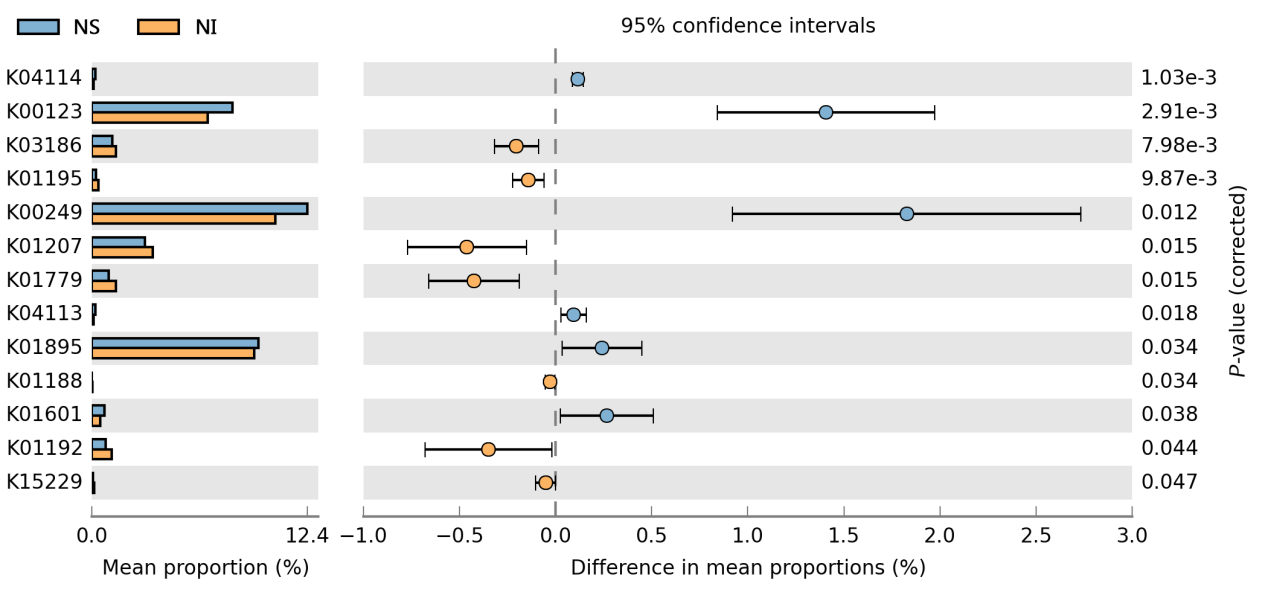


**Fig. S4 Proportional abundance of predicted soil C‑cycling genes across treatments.** Comparisons within severely degraded soil: (a) SC vs. SS, (b) SC vs. SI, (c) SS vs. SI. (d) Comparisons within moderately degraded soil (MS vs. MI). (e) Comparisons within non‑degraded soil (NS vs. NI). The x-axis shows the difference in mean proportional abundance (%) between paired treatments, with 95% confidence intervals displayed as horizontal error bars. The y-axis lists individual carbon-cycling genes (KO IDs). Differences between paired treatments were assessed using the Wilcoxon rank‑sum test, and *P*‑values were adjusted for multiple testing using the false discovery rate (FDR) method. SC, severely degraded soil, control; SS, severely degraded soil with straw addition; SI, severely degraded soil with straw combined with microbial inoculants; MS, moderately degraded soil with straw addition; MI, moderately degraded soil with straw combined with microbial inoculants; NS, non-degraded soil with straw addition; NI, non-degraded soil with straw combined with microbial inoculants.

**a**


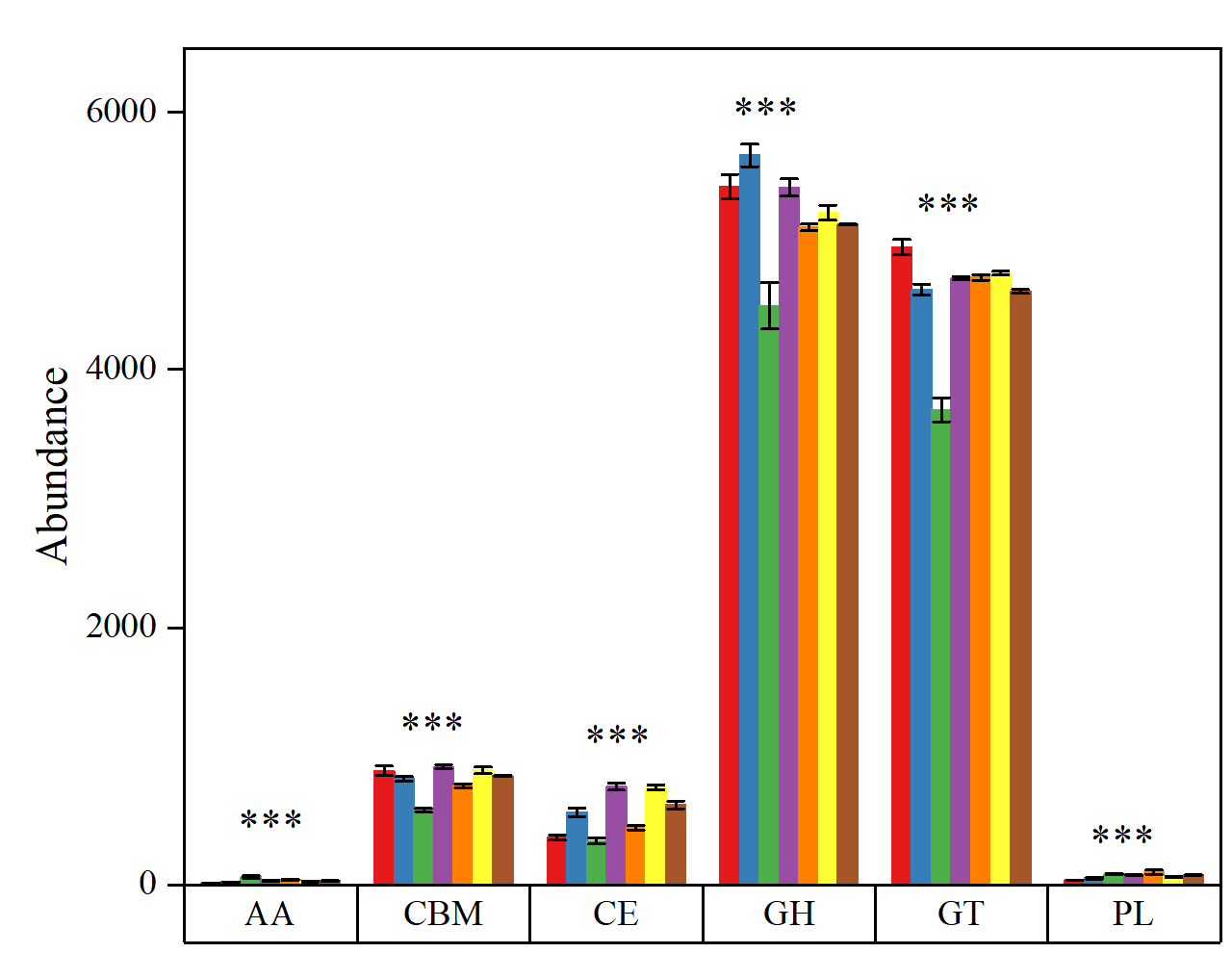


**b**


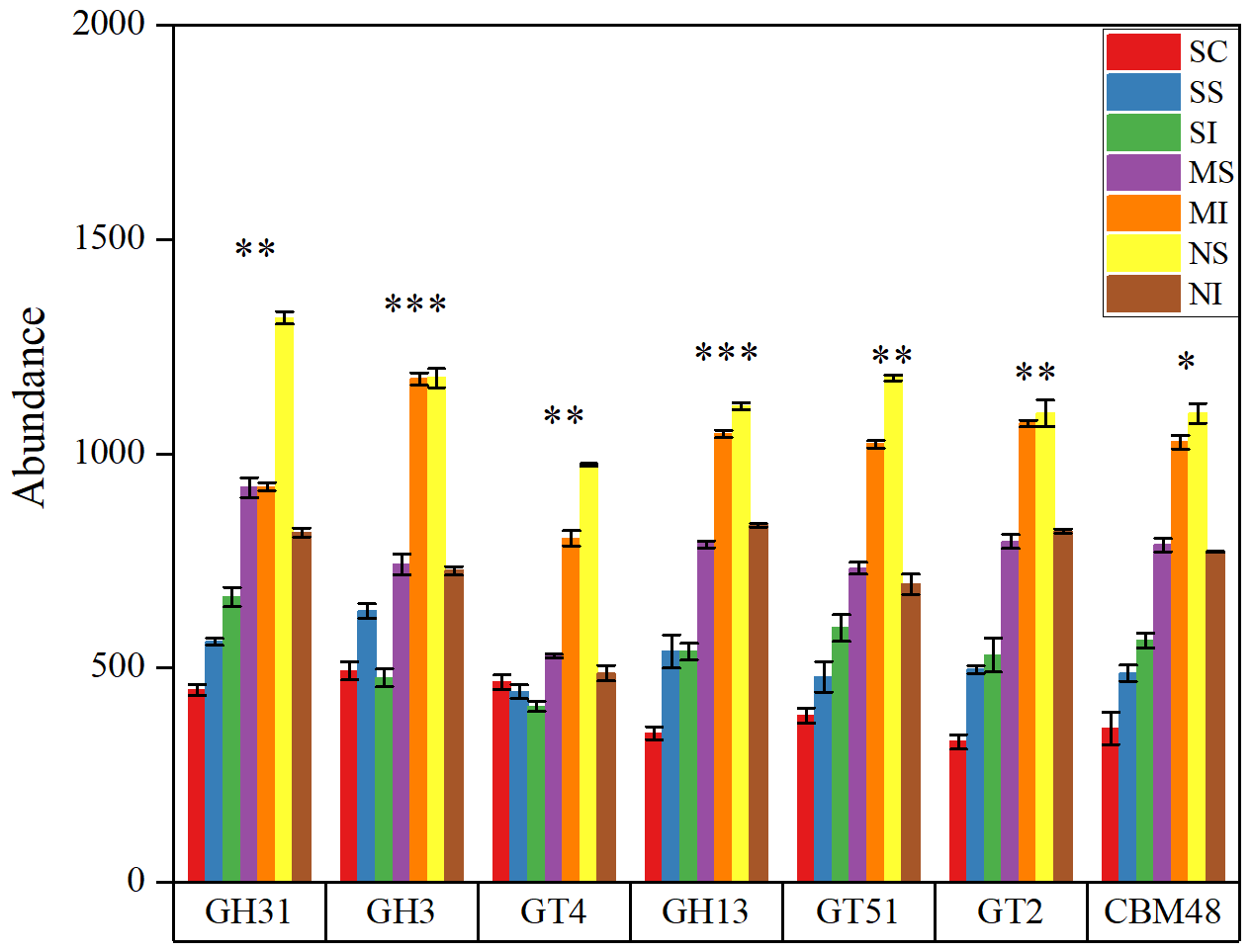


**Fig. S5 Differential abundance of CAZy families related to C cycling across treatments.** (a) Abundance of total CAZy functions. (b) Abundance of the top 7 individual CAZy families. Sample data are presented as mean±SE, n=3. The asterisk (*,**,***) indicates the significant (*P*< 0.05, *P*< 0.01, *P* < 0.001) differences among the seven treatments. AA:auxiliary activities. CBM:carbohydrate-binding modules. CE:carbohydrate esterases. GH: glycoside hydrolases. GT: glycosyl transferase. PL:polysaccharide lyases. GH31:alpha-glucosidase. GH3:beta-glucosidase. GT4:sucrose synthase. GH13: alpha-amylase. GT51:murein polymerase. GT2:cellulose synthase. CBM48:modules of approx. SC, severely degraded soil, control; SS, severely degraded soil with straw addition; SI, severely degraded soil with straw combined with microbial inoculants; MS, moderately degraded soil with straw addition; MI, moderately degraded soil with straw combined with microbial inoculants; NS, non-degraded soil with straw addition; NI, non-degraded soil with straw combined with microbial inoculants.

**a**


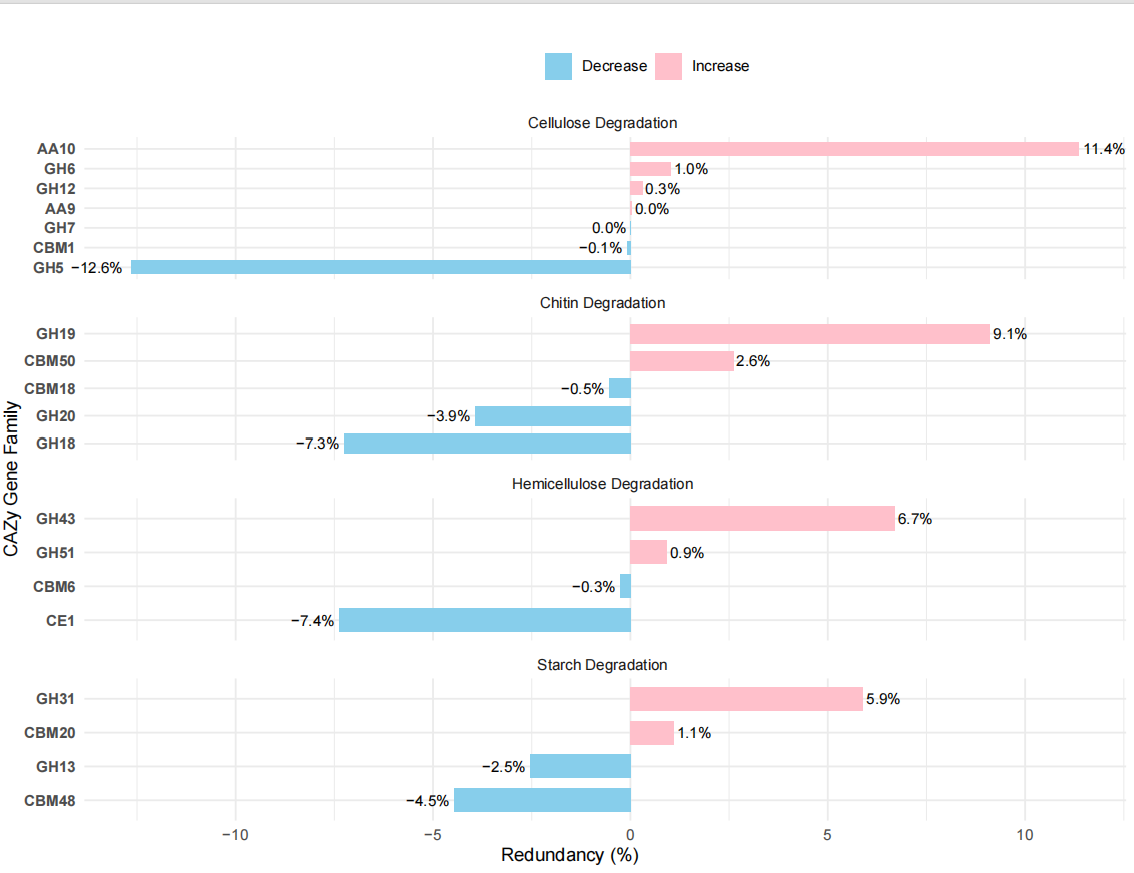

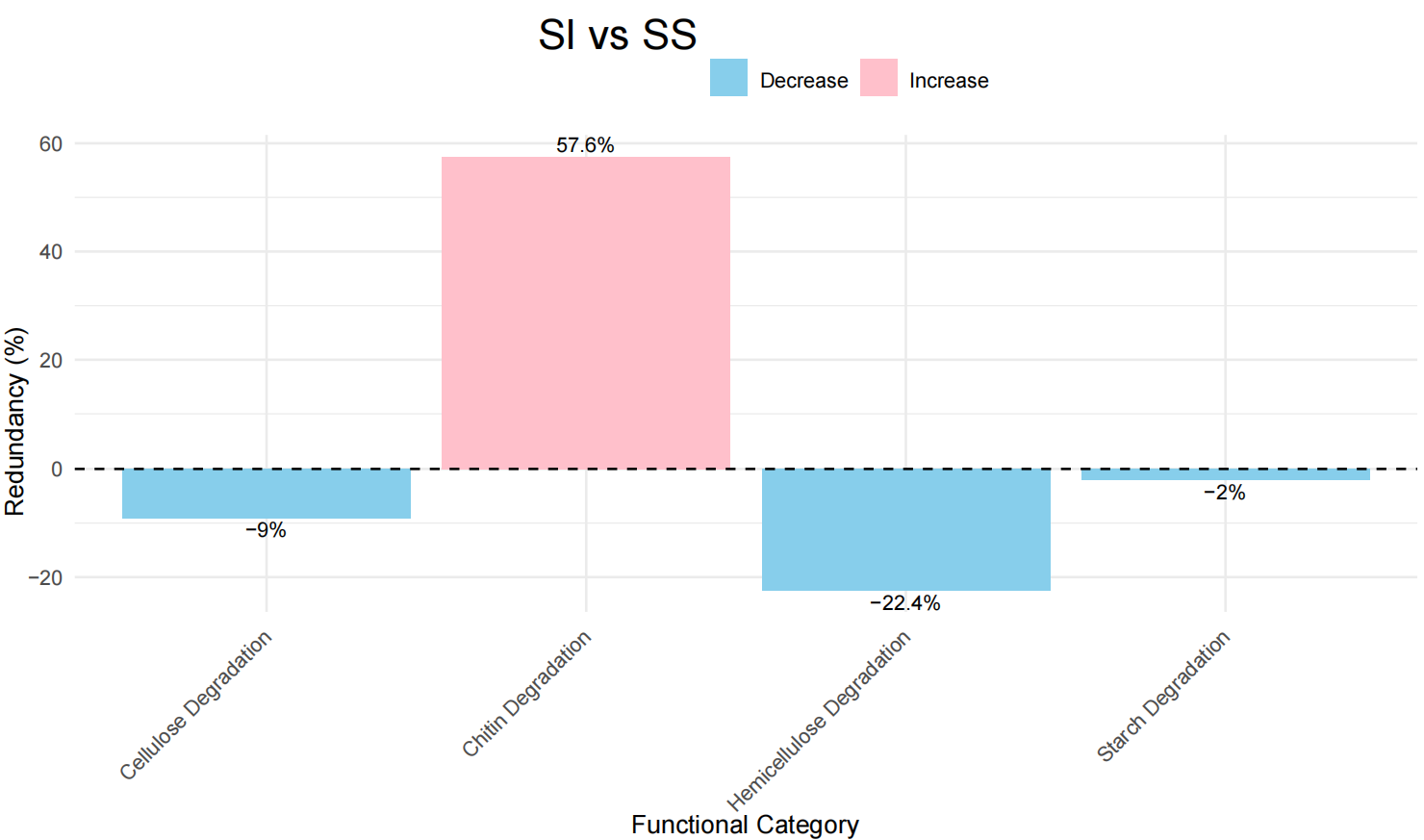


**b**


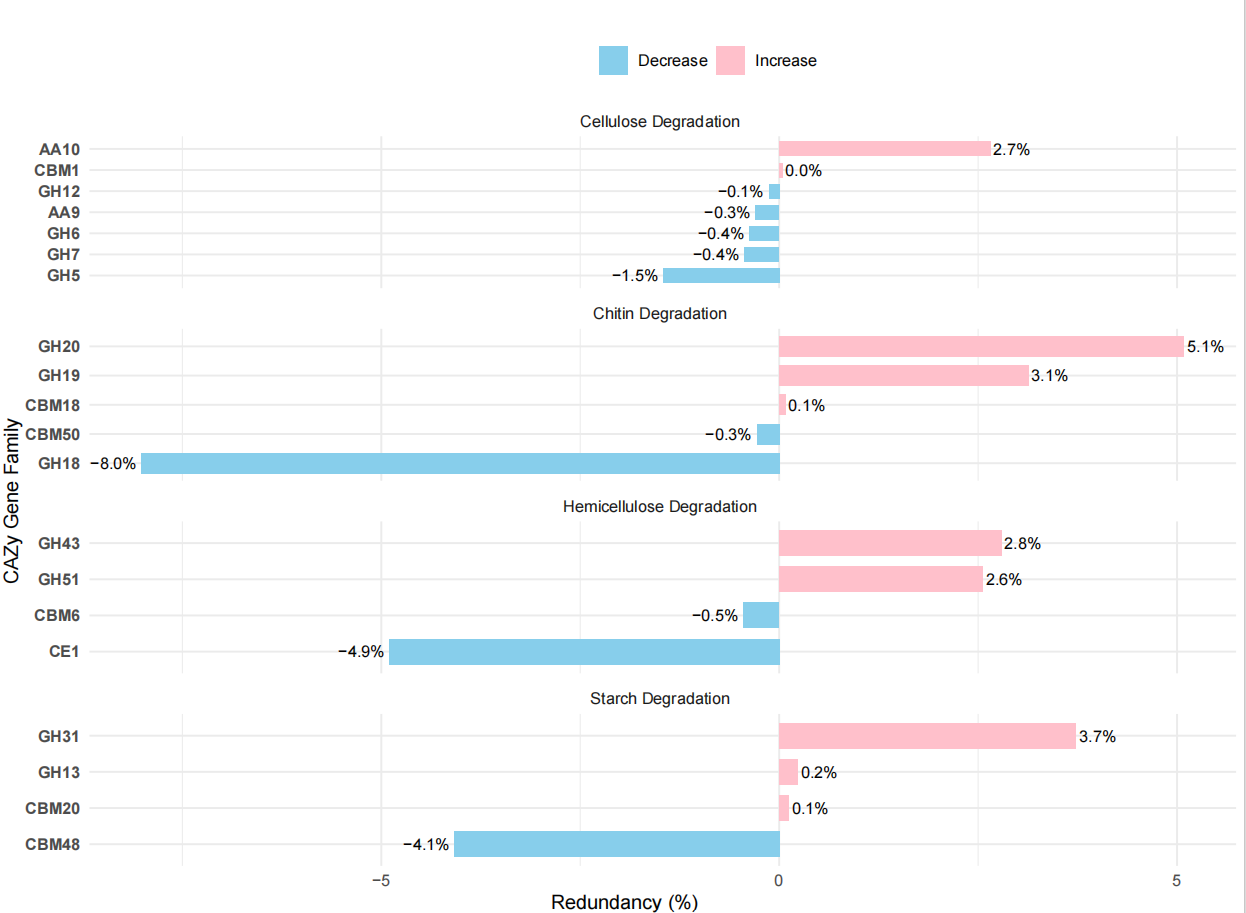


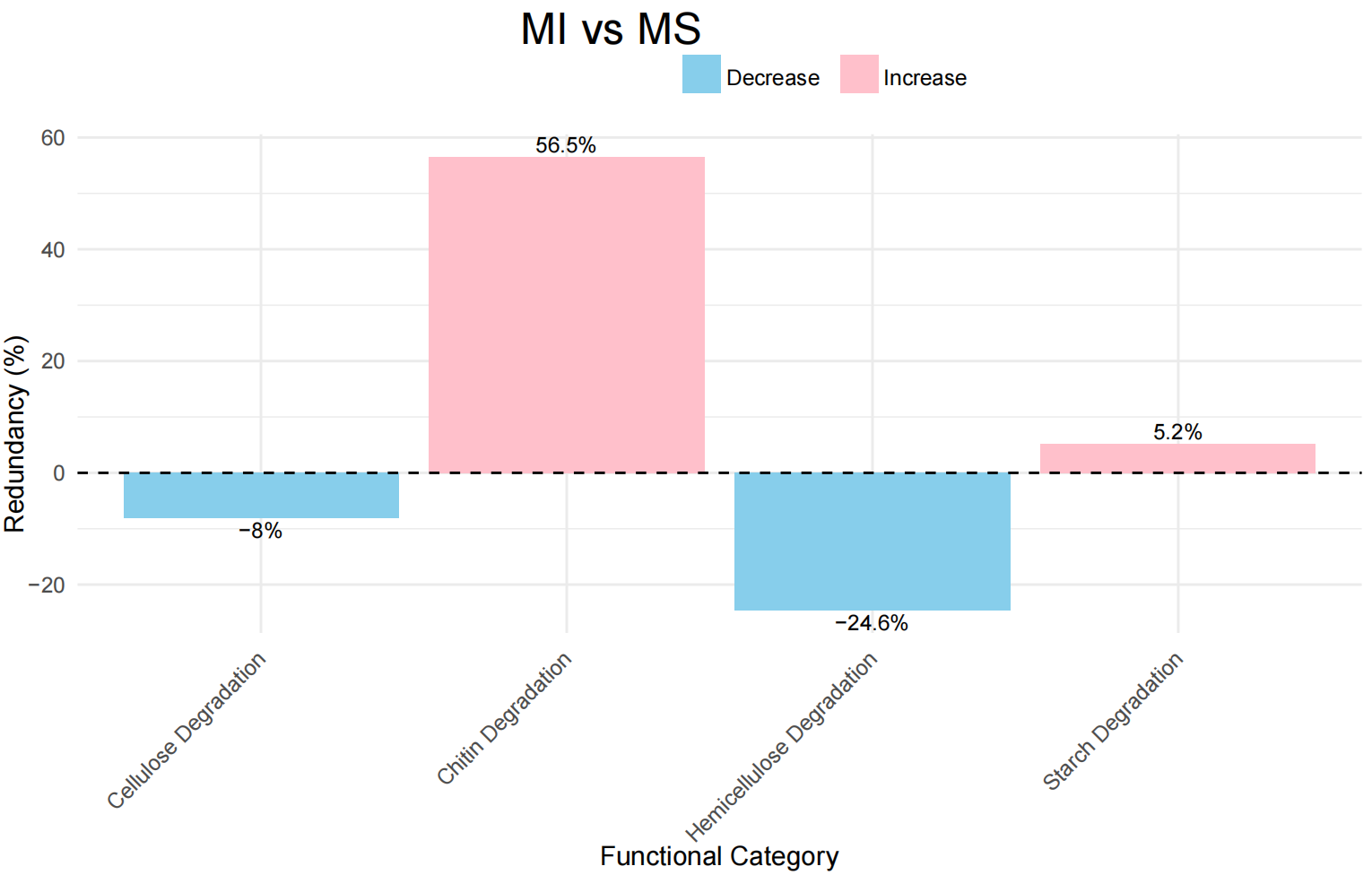


**c**


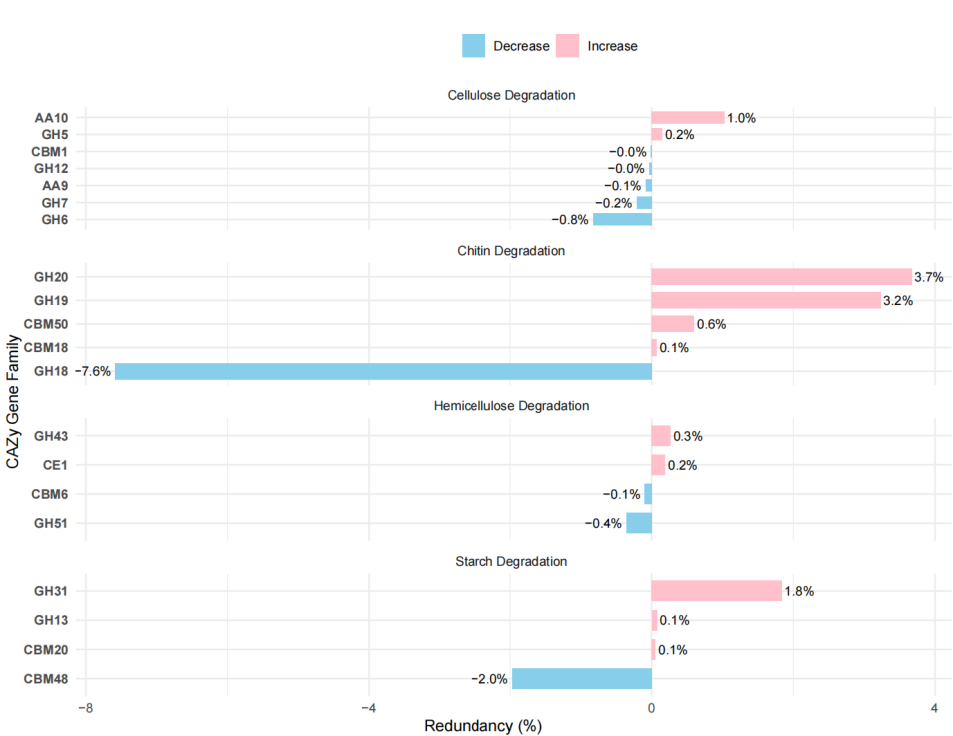


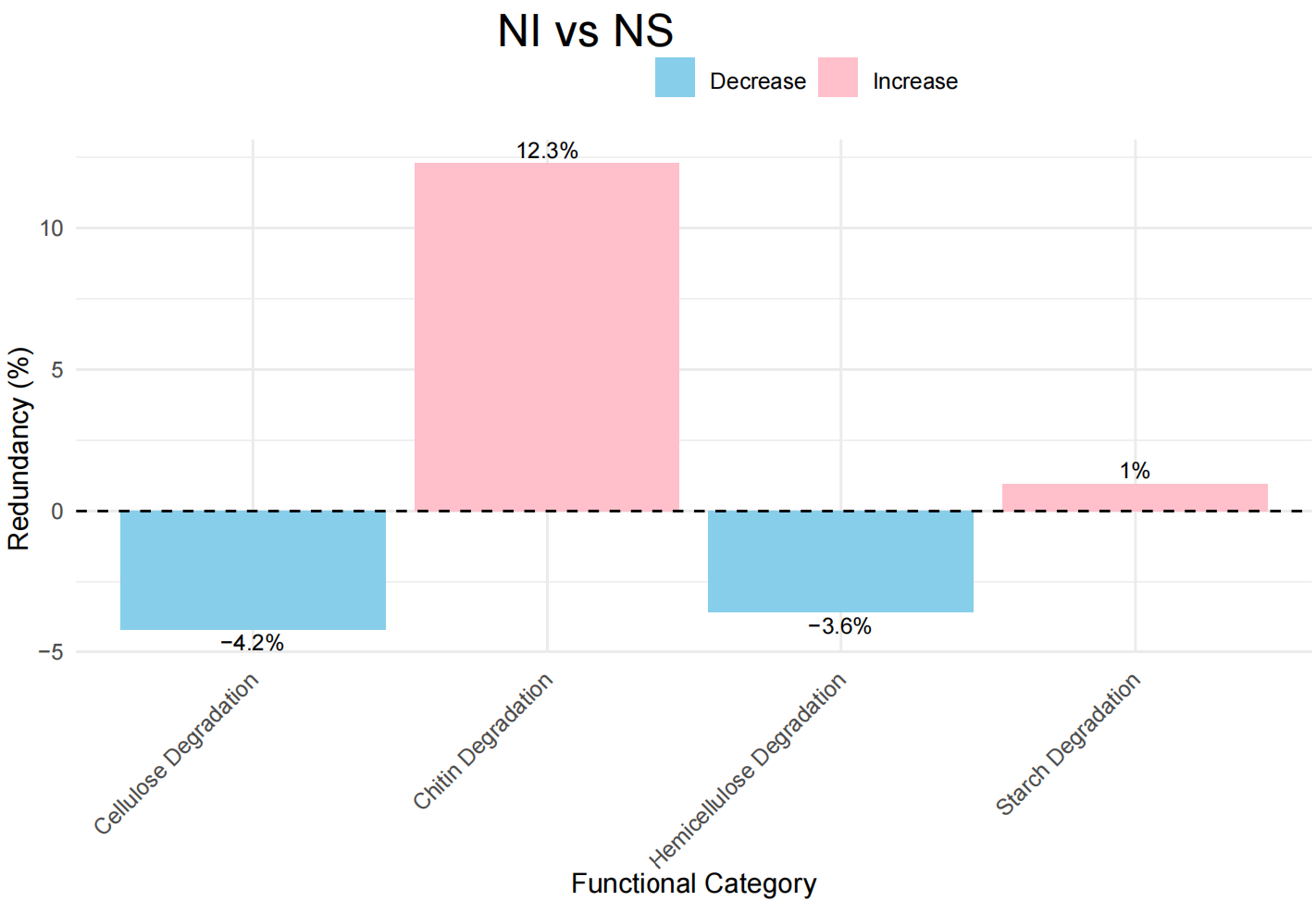


**Fig. S6 Effects of microbial inoculants on the redundancy of functional genes for carbon degradation in different degraded cinnamon soil.** (a) Severely degraded soil. (b) Moderately degraded soil. (c) Non-degraded soil. SS, severely degraded soil with straw addition; SI, severely degraded soil with straw combined with microbial inoculants; MS, moderately degraded soil with straw addition; MI, moderately degraded soil with straw combined with microbial inoculants; NS, non-degraded soil with straw addition; NI, non-degraded soil with straw combined with microbial inoculants. Cellulose Degradation: AA9, copper-dependent polysaccharide monooxygenase; AA10, copper-dependent polysaccharide monooxygenase; CBM1, carbohydrate-binding module that binds to cellulose; GH5, endo-beta-1,4-glucanase; GH6, cellobiohydrolase; GH7, endo/exo-β-1,4-glucanase; GH12, beta-1,3-1,4-glucanase. Chitin Degradation: CBM18, carbohydrate-binding module that binds to chitin; CBM50, carbohydrate-binding module that binds to chitin; GH18, chitinase; GH19, chitinase; GH20, beta-6-SO_3_-N-acetylglucosaminidase. Hemicellulose Degradation: CBM6, carbohydrate-binding module that binds to hemicellulose; CE1, carboxylesterase; GH43, beta-xylosidase; GH51, endo-beta-1,4-xylanase. Starch Degradation: CBM20, carbohydrate-binding module that binds to starch; CBM48, modules of approx; GH13, alpha-amylase; GH31:alpha-glucosidase.


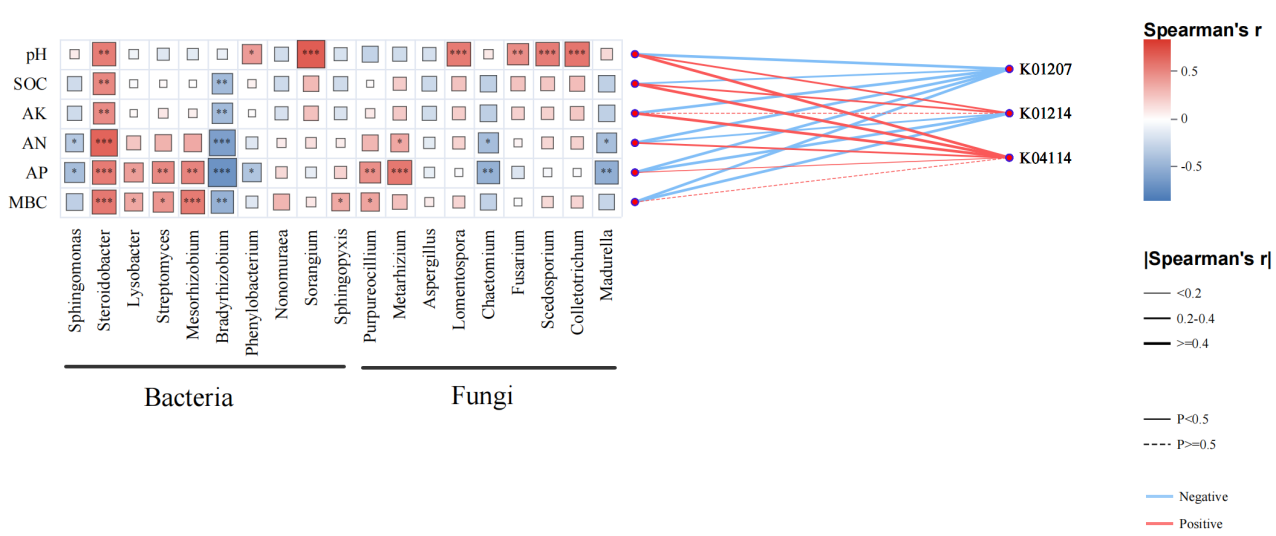


**Fig. S7 Correlations between key C-cycling genes, soil properties, and microbial taxa (genus level).** The analysis focuses on the differential response of specific genes between paired treatments: gene K01214 in severely degraded soil (SI vs. SS), gene K01207 in moderately degraded soil (MI vs. MS), and gene K04114 in non-degraded soil (NI vs. NS). Edges represent significant Spearman correlations, with line color indicating the sign of the correlation coefficient (solid: positive; dashed: negative) and line width proportional to the absolute value of Spearman's r. *, *P* < 0.05; **, *P* < 0.01, ***, *P* < 0.001. SS, severely degraded soil with straw addition; SI, severely degraded soil with straw combined with microbial inoculants; MS, moderately degraded soil with straw addition; MI, moderately degraded soil with straw combined with microbial inoculants; NS, non-degraded soil with straw addition; NI, non-degraded soil with straw combined with microbial inoculants.

1. [↑](#footnote-ref-0)
2. [↑](#footnote-ref-1)
